# Hydrophilic Photocrosslinkers as a Universal Solution to Endow Water Affinity to a Polymer Photocatalyst for an Enhanced Hydrogen Evolution Rate

**DOI:** 10.1002/advs.202309786

**Published:** 2024-05-17

**Authors:** Sanghyeok An, Kyeong‐Jun Jeong, Syed Zahid Hassan, Gayoung Ham, Seonghyeon Kang, Juhyeok Lee, Hyeonjong Ma, Jieun Kwon, Sang Young Jeong, Jiwoong Yang, Han Young Woo, Han‐Hee Cho, Hyojung Cha, Chang Yun Son, Dae Sung Chung

**Affiliations:** ^1^ Department of Chemical Engineering Pohang University of Science and Technology (POSTECH) Pohang 37673 Republic of Korea; ^2^ Department of Chemistry Pohang University of Science and Technology (POSTECH) Pohang 37673 Republic of Korea; ^3^ Department of Energy Convergence and Climate Change Kyungpook National University Daegu 41566 Republic of Korea; ^4^ Department of Energy Science and Engineering Daegu Gyeongbuk Institute of Science and Technology (DGIST) Daegu 42988 Republic of Korea; ^5^ Department of Chemistry Korea University Seoul 02841 Republic of Korea; ^6^ Department of Materials Science and Engineering Ulsan National Institute of Science and Technology (UNIST) Ulsan 44919 Republic of Korea; ^7^ Department of Hydrogen & Renewable Energy Kyungpook National University Daegu 41566 Republic of Korea

**Keywords:** charge carrier stabilization, exciton dynamics, hydrogen evolution, hydrophilic photocrosslinkers, molecular dynamics simulations, photocatalytic performance, polymer photocatalysts

## Abstract

A universal approach for enhancing water affinity in polymer photocatalysts by covalently attaching hydrophilic photocrosslinkers to polymer chains is presented. A series of bisdiazirine photocrosslinkers, each comprising bisdiazirine photophores linked by various aliphatic (CL‐R) or ethylene glycol‐based bridge chains (CL‐TEG), is designed to prevent crosslinked polymer photocatalysts from degradation through a safe and efficient photocrosslinking reaction at a wavelength of 365 nm. When employing the hydrophilic CL‐TEG as a photocrosslinker with polymer photocatalysts (F8BT), the hydrogen evolution reaction (HER) rate is considerably enhanced by 2.5‐fold compared to that obtained using non‐crosslinked F8BT photocatalysts, whereas CL‐R‐based photocatalysts yield HER rates comparable to those of non‐crosslinked counterparts. Photophysical analyses including time‐resolved photoluminescence and transient absorption measurements reveal that adding CL‐TEG accelerates exciton separation, forming long‐lived charge carriers. Additionally, the in‐depth study using molecular dynamics simulations elucidates the dual role of CL‐TEG: it enhances water penetration into the polymer matrix and stabilizes charge carriers after exciton generation against undesirable recombination. Therefore, the strategy highlights endowing a high‐permittivity environment within polymer photocatalyst in a controlled manner is crucial for enhancing photocatalytic redox reactivity. Furthermore, this study shows that this hydrophilic crosslinker approach has a broad applicability in general polymer semiconductors and their nanoparticulate photocatalysts.

## Graphical abstract

In this work, a universal strategy is proposed to impart water affinity to common organic photocatalysts by forming 3D hydrophilic crosslinking structures between polymer‐semiconductor chains using hydrophilic photocrosslinking agents. This enables organic photocatalysts to have a high permeability environment in a controlled manner, which is found to be crucial for enhancing photocatalytic redox reactivity.

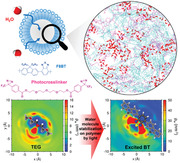

## 1 Introduction

The rapid depletion of fossil fuels and the environmental pollution resulting from carbon emissions have significantly impacted the global economy and natural environment, prompting a strong demand for cleaner energy sources.^[^ ^1^, ^2^ ^]^ Photocatalytic water splitting is a promising, sustainable technology to address these challenges by producing solar‐driven H_2_ in economically feasible manner.^[^ ^3^, ^4^ ^]^ Traditional photocatalysts such as TiO_2_ and SrTiO_3_ can directly convert solar energy into hydrogen by splitting water. However, modifying their optical and energetic properties is limited by the inherent constraints in altering inorganic semiconductors.^[^ ^5^, ^6^, ^7^ ^]^ In recent years, organic semiconductors have emerged as favorable alternatives to their inorganic counterparts in solar‐driven photocatalytic water splitting by leveraging their unique advantages, such as a diverse range of chemical structures and compositions and the tunability of energy levels.^[^ ^8^, ^9^, ^10^, ^11^, ^12^, ^13^ ^]^ Moreover, these organic semiconductors can absorb a broader spectrum of solar radiation while maintaining the necessary energy‐level alignment for driving redox reactions. These reactions include the hydrogen evolution reaction (HER), oxygenation reactions, and alternative redox processes for the production of solar fuels and chemical feedstocks.^[^ ^14^, ^15^, ^16^, ^17^, ^18^ ^]^ Indeed, various polymer photocatalysts have been investigated, including graphitic carbon nitride,^[^ ^19^ ^]^ linear conjugated polymers,^[^ ^20^ ^]^ conjugated microporous polymers,^[^ ^21^ ^]^ and covalent organic frameworks.^[^ ^22^ ^]^

Despite these advantages, polymeric photocatalytic materials often exhibit less hydrophilicity than their inorganic counterparts, primarily owing to large aromatic groups and long aliphatic side chains.^[^ ^23^ ^]^ Given that photocatalytic redox reactions occur in aqueous media, the hydrophobic nature of organic semiconductors can lead to issues with water affinity, thereby limiting their HER and/or OER efficiency. Various strategies have been developed to overcome these challenges to increase the hydrophilicity of polymer photocatalysts. These include integrating polar backbone units with sulfone groups and adding hydrophilic side chain units, such as ethylene glycol.^[^ ^24^, ^25^ ^]^ Kosco et al. reported a significant enhancement in HER performance by introducing ethylene glycol side chains into traditional polymer photocatalysts such as IDTBT, F8BT, and PTB7‐Th.^[^ ^26^ ^]^ Similarly, Woods et al. demonstrated that replacing alkyl side chains with glycol side chains improved the lifetimes of photogenerated polaronic states.^[^ ^23^ ^]^ Huang et al. enhanced the performance of polymer photocatalysts by linking a conjugated polymer backbone with glycol side‐chain bridges.^[^ ^27^ ^]^ These enhancements can be ascribed to the augmented water‐uptake properties afforded by an increased hydrophilicity, leading to improved permittivity. In organic semiconductors, challenges such as the relatively high exciton binding energy, attributed to the Frenkel exciton property, and the propensity for recombination of once‐separated charge carriers are notable disadvantages for their application as photocatalysts.^[^ ^6^ ^]^ However, the introduction of ethylene glycol side chains, which elevate water affinity and thus permittivity within organic semiconductors in aqueous environments, can tackle these challenges by providing a reduced exciton binding energy and diminished susceptibility to recombination. Consequently, devising a method for uniformly and universally integrating ethylene glycol functional groups into polymer photocatalysts is highly promising for advancing photocatalytic performance of polymer photocatalysis.

**Scheme 1:**
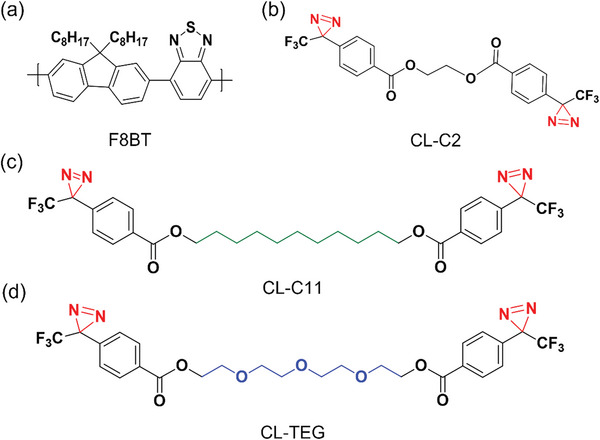
Chemical structures of a conjugated polymer a) F8BT and photocrosslinkers, b) CL‐C2, c) CL‐C11, and d) CL‐TEG. CL‐C11 was synthesized to have the same chain length as CL‐TEG.

Diazirine photophores are known in chemical biology for their capacity to generate reactive carbene by releasing a N_2_ moiety under UV‐A light exposure (320–400 nm). This process facilitates a rapid insertion reaction with adjacent functional groups, including O─H, N─H, or C─H bonds. Furthermore, singlet carbenes can be transferred via intersystem crossing to form triplet carbenes, thereby enhancing crosslinking via hydrogen abstraction and radical recombination.^[^ ^28^, ^29^ ^]^ Indeed, diazirine is a well‐established and highly stable photophore, efficiently generating carbenes upon UV‐A exposure. Additionally, it remains inert to common nucleophiles and electrophiles without UV light. This stability facilitates long‐term storage at room temperature in the dark. In contrast to azide‐containing crosslinkers that incorporate nitrogen atoms into the side chains of conjugated polymers, crosslinking reactions involving diazirine‐based crosslinkers do not introduce nitrogen atoms.^[^ ^30^ ^]^ This contrasts with azides, where the nitrogen atom in nitrene forms an amine post‐photocrosslinking, imparting essential characteristics to the system. However, the design of novel diazirine‐based crosslinkers must consider several critical aspects: 1) The propensity of diazirine for isomerization to diazo leads to undesirable side reactions alongside carbene insertion at the C─H bond. Incorporating a CF_3_ group can effectively slow down diazo formation, thereby reducing unwanted side reactions. 2) The linear configuration of the two diazirine groups in the crosslinker, interconnected via straight alkyl chains without zigzag configurations, is demanded. This structure significantly improves crosslinking efficiency with randomly oriented alkyl chains in a conjugated polymer. 3) A Linker needs to incorporate an electron‐withdrawing benzoate group so as to enable the formation of more stable carbenes and thus reduce the likelihood of generating undesired byproducts.^[^ ^31^, ^32^ ^]^ In this context, we introduce a synthetically straightforward and universally applicable hydrophilic crosslinking approach to augment water affinity in polymer photocatalysts. We designed and synthesized a series of bisdiazirine photocrosslinkers, featuring bisdiazirine as the photophore and various bridging components. These include ethyl, undecyl, and tetraethylene glycol, designated CL‐C2, CL‐C11, and CL‐TEG, respectively (**Scheme** **1**). Here CL‐C11 was synthesized to match the alkyl chain length of CL‐TEG. Integrating bisdiazirine enabled the photocrosslinking reaction under UV irradiation at 365 nm, a milder condition than those associated with traditional azide photocrosslinkers that necessitate UV irradiation at 254 nm.^[^ ^31^, ^32^ ^]^ Photocrosslinking with CL‐TEG efficiently incorporated TEG functional groups into the F8BT photocatalyst interior, yielding a more than 2.5‐fold enhancement in the HER rate. Our analyses, encompassing time‐resolved photoluminescence (TR‐PL) and transient absorption (TA) spectroscopy, indicate that CL‐TEG photocrosslinking not only expedites exciton separation but also increases the population of long‐lived charge carriers within the F8BT photocatalysts. Moreover, molecular dynamics simulations corroborated the marked improvement in water accessibility to the F8BT polymer chains following CL‐TEG introduction. Furthermore, we demonstrated that the beneficial effects of hydrophilic photocrosslinking strategies are extendable to other polymer photocatalysts, presenting promising prospects for future research in this domain.

## 2 Results and Discussion

### 2.1 Synthesis of Nanoparticles with Photocrosslinkers

We systematically designed and synthesized three novel photocrosslinkers based on diazirine photophores by varying the bridge chains between the photophores: ethane‐1,2‐diyl bis(4‐(3‐trifluoromethyl)‐3H‐diazirin‐3‐yl)benzoate (CL‐C2), octane‐1,8‐diyl bis(4‐(3‐trifluoromethyl)‐3H‐diazirin‐3‐yl)benzoate (CL‐C11) and ((oxybis(ethane‐2,1‐diyl))bis(oxy))bis(ethane‐2,1‐diyl) bis(4‐(3‐(trifluoromethyl)‐3H‐diazirin‐3‐yl)benzoate) (CL‐TEG), as depicted in Scheme 1. The three crosslinkers were precisely engineered to enable effective photocrosslinking within polymer nanoparticles. While the CL‐C2, characterized by a shorter ethylene‐based alkyl chain, serves as the control crosslinker, the CL‐C11 variant exhibits more pronounced nonpolar properties due to its undecane bridge. Notably, the CL‐TEG variant is distinguished by high polarity stemmed from its highly polar tetraethylene glycol bridge, which is advantageous for preparing a broad range of photocatalytic polymer nanoparticles dispersed in aqueous electrolytes. Moreover, it can play a crucial role in promoting water transport to the surface of polymer nanoparticles, consequently amplifying their photocatalytic efficacy.

The synthetic route for these diazirine‐based crosslinkers is depicted in Figure S1 (Supporting Information). In specific, an intermediate, 4‐[3‐(Trifluoromethyl)‐3H‐diazirin‐3‐yl]benzoic acid, was synthesized following the method described by Sakurai et al^[^ ^33^, ^34^ ^]^ and then coupled with ethylene glycol, 1,11‐undecanediol, and tetraethylene glycol to produce the crosslinkers CL‐C2, CL‐C11, and CL‐TEG, respectively. Thus, the three crosslinker variants, CL‐C2, CL‐C11, and CL‐TEG, share a common functional group, yet they exhibit variations in their bridge chain lengths. Their molecular structures were verified using ^1^H, ^13^C, and ^19^F NMR spectroscopic analyses, as shown in Figures S2–S10 (Supporting Information). The solution‐phase UV absorption spectra of the three photocrosslinkers, presented in Figure S11 (Supporting Information), display similar absorption peaks at 365 nm with a practical molar absorptivity of 553 cm^−1^ M^−1^. This specific absorptivity is sufficient to trigger the decomposition of diazirine into carbene under UV‐A light irradiation (365 nm), as evidenced by the UV–vis spectra (Figure S11b, Supporting Information). Consequently, successful crosslinking of polymer nanoparticles is expected using the newly designed photocrosslinkers. All three crosslinkers demonstrate solubility in a wide range of common organic organic solvents.(Figure S12, Supporting Information).

### 2.2 Characterization of Nanoparticles

**Figure 1:**
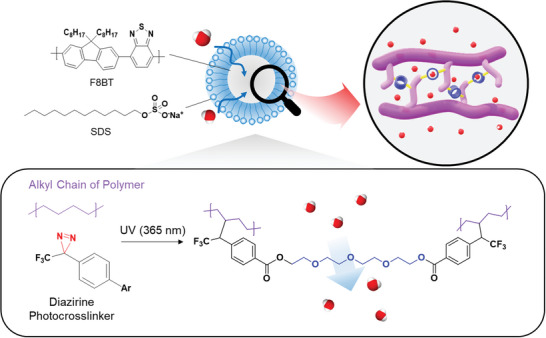
Schematic drawing of a photocrosslinked conjugated polymer nanoparticle fabrication process (via mini‐emulsion method), including the chemical structures of a conjugated polymer photocatalysts (F8BT), diazirine crosslinker (CL‐TEG), and a surfactant (SDS). The alkyl side chains of F8BT are photocrosslinked with the diazirine crosslinker (CL‐TEG) by 365 nm UV light.

Previous research has utilized various techniques, such as nano‐precipitation and mini‐emulsion, to fabricate polymer photocatalyst nanoparticles. In the nano‐precipitation method, polystyrene‐polyethylene glycol‐carboxyl (PS‐PEG‐COOH) is typically used as a polymeric surfactant for nanoparticle synthesis.^[^ ^20^ ^]^ However, these methods often limit dispersion stability in aqueous media. When the side chain of a polymeric surfactant interacts with a hydrophilic environment, such as the aqueous/organic interface, it tends to adopt an unstable conformation, which frequently leads to aggregation.^[^ ^8^ ^]^ In contrast, mini‐emulsion methods employ oligomeric surfactants such as 2‐(3‐thienyl)ethyloxybutylsulfonate (TEBS) and sodium dodecyl sulfate (SDS) to disperse hydrophobic polymer photocatalysts in an aqueous medium.^[^ ^35^ ^]^ In this study, we used SDS as the surfactant for nanoparticle synthesis owing to its proven effectiveness in enhancing HER performance compared to that of TEBS for F8BT (**Figure** **1**).^[^ ^32^, ^35^ ^]^

**Figure 2:**
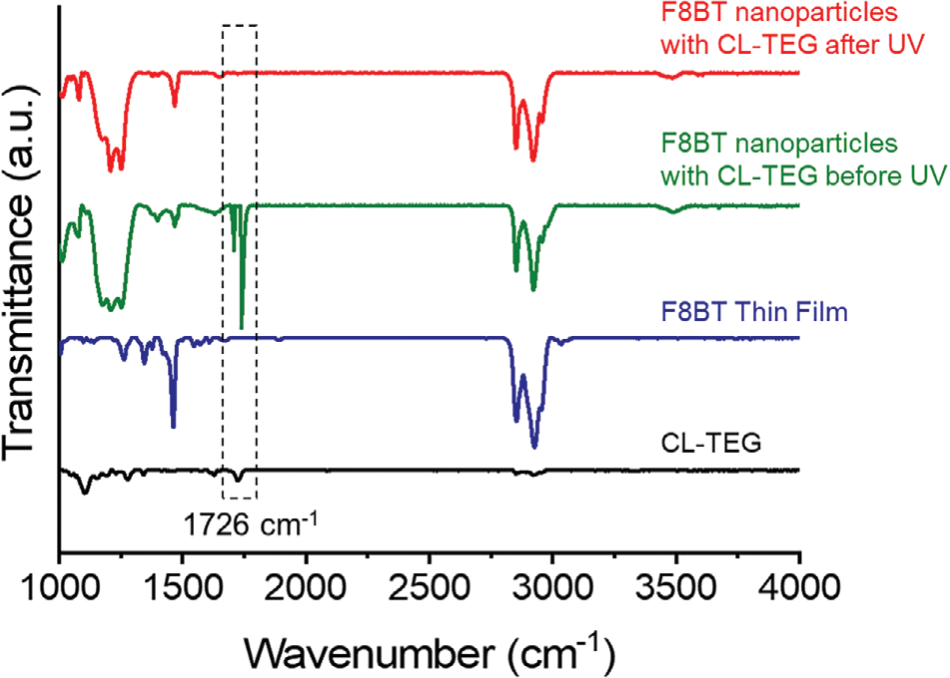
Solid‐state FT‐IR spectra of F8BT nanoparticles and CL‐TEG: photocrosslinked F8BT nanoparticles with CL‐TEG (red); F8BT and CL‐TEG nanoparticles without UV irradiation (green); neat F8BT thin film (blue); neat CL‐TEG thin film (black). For F8BT nanoparticles prepared with CL‐TEG, diazirine stretching bands at 1726 cm^−1^ vanish after 365 nm UV light irradiation. All nanoparticles and thin films of F8BT and CL‐TEG were drop‐casted on Si wafers. The F8BT and crosslinkers were dissolved in chloroform.

To validate the crosslinking within the nanoparticles, we examined the Fourier‐transform infrared (FT‐IR) spectra of the photocrosslinked photocatalysts. As depicted in **Figure** **2**, the polymer blends containing crosslinking agents before photocrosslinking exhibit diazirine stretching bands characterized by an N═N stretching frequency (≈1726 cm^−1^) whereas the pristine conjugated polymer displays no distinct bands corresponding to the characteristic N═N stretching frequency. Interestingly, the diazirine stretching band for the photocrosslinked conjugated polymer blend with the CL‐TEG was clearly vanished after the crosslinking under the 365 nm UV light irradiation. As illustrated in Figure S13 (Supporting Information), other photocrosslinkers (CL‐C2 and CL‐C11) show similar band changes. Indeed, the IR bands of the pristine polymer and those of the photocrosslinked polymer blend revealed no significant differences after the crosslinking. The analysis of the IR spectra of the thin films substantiates the successful crosslinking between the nanoparticles. Additionally, NMR analysis was conducted to demonstrate the occurrence of the C–H insertion reaction in the crosslinker blended with polymers. To thoroughly investigate this reaction, we selected cyclohexane as the model compound. This is because cyclohexane has only one proton peak in its alkyl region in ^1^H NMR, facilitating the analysis of the 12 protons involved during the C–H insertion reaction under UV light irradiation.^[^ ^34^ ^]^ We included ^1^H NMR and ^19^F NMR spectra in the Supporting Information (Figures S15 and S16, Supporting Information), both before and after UV irradiation, demonstrating successful carbene insertion reactions with cyclohexane present in the system. As depicted in Figure S14 (Supporting Information), cyclohexane underwent C–H insertion from CL‐C11 and the formation of CL‐C11‐cyclohexane complex via a photochemically induced C–H insertion reaction. With the FT‐IR analysis, which showed the removal of N_2_ after diazirine breakdown, NMR spectroscopy confirmed the reaction of photochemically induced C–H insertion. This supported the evidence for the crucial role of C–H insertion, which happened when diazirine forms carbene under UV light exposure. The method for preparing the NMR sample is detailed in the experimental procedures section.

**Figure 3:**
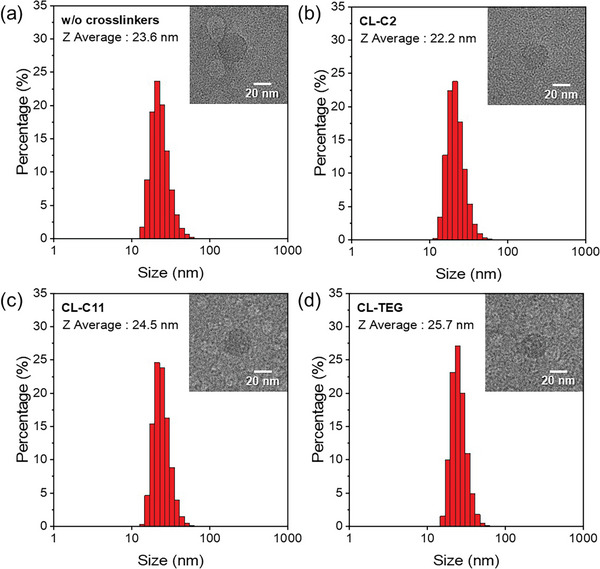
Measured sizes of F8BT nanoparticles via dynamic light scattering (DLS) are presented: a) without crosslinker and photocrosslinked with b) CL‐C2, c) CL‐C11, and d) CL‐TEG. Insets feature liquid‐phase transmission electron microscopy (LP‐TEM) images. The samples were photocrosslinked via exposure to 365 nm UV light for 30 min.

Dynamic light scattering (DLS) analysis was conducted on the nanoparticles photocrosslinked using various agents, revealing similar hydrodynamic radii ranging from 20 to 30 nm irrespective of the presence of photocrosslinkers, as shown in **Figure** **3**. We further investigated the morphology of these nanoparticles using liquid‐phase transmission electron microscopy (LP‐TEM).^[^ ^36^ ^]^ Consistent with the DLS results, the LP‐TEM images show spherical nanoparticles with diameters slightly exceeding 20 nm in all cases. Notably, the nanoparticle porosity or density appears unaffected by the presence or types of crosslinker used. Consequently, the HER enhancement attributed to the hydrophilic crosslinker (CL‐TEG), to be discussed subsequently, is not linked to any morphological alterations induced by crosslinking.

### 2.3 Hydrogen Evolution Rate

**Figure 4:**
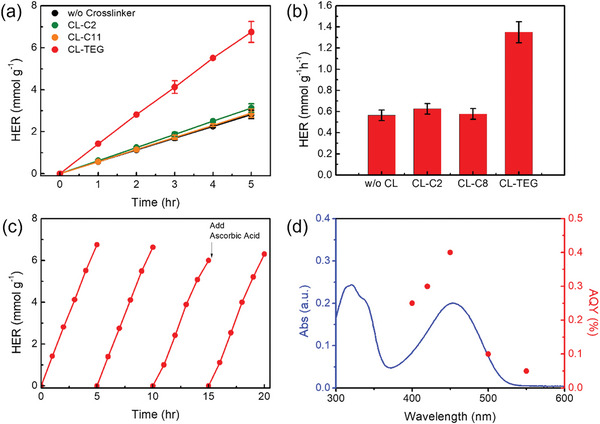
a) Comparison of the photocatalytic hydrogen evolution activities of F8BT prepared without photocrosslinker and with CL‐C2, CL‐C11, and CL‐TEG using ascorbic acid (pH 4) as a sacrificial agent (*λ* > 420 nm). b) Hydrogen evolution rate (HER) for each sample (the average of 5 h across five repeated runs). c) Photocatalytic stability tests up to 20 h for optimized F8BT nanoparticles photorosslinked via CL‐TEG. d) Apparent quantum yield (AQY) of F8BT photocrosslinked via CL‐TEG at 400, 420, 450, 500, and 550 nm along with the absorbance spectrum of a 0.2 m (pH 4) solution of ascorbic acid. The error bars were obtained from the five standard deviation derived from five repeated trials.

The photocatalytic efficiency of the polymer photocatalysts was assessed under simulated solar illumination (Xenon Lamp, 70 mW cm^−2^, *λ* > 420 nm Bandpass filter). We employed ascorbic acid (pH 4) buffered with a NaOH solution as a sacrificial hole scavenger for this evaluation. The HER performance was measured via gas chromatography using a Luer‐Lock gas syringe. As indicated in **Figure** **4**, the photocrosslinked F8BT with CL‐TEG demonstrates an average HER rate of 1350 µmol h^−1^ g^−1^ over 5 h of illumination, representing an ≈2.5‐fold increase in performance compared to that of the non‐crosslinked pristine F8BT. F8BT nanoparticles photocrosslinked with CL‐C2 and CL‐C11 show only modest performance improvements, achieving rates of 626 and 577 µmol g^−1^ h^−1^, respectively, which are 1.4 and 1.2 times higher compared to the pristine case.

F8BT nanoparticles photocrosslinked with CL‐C2 and CL‐C11 showed only modest performance improvement, achieving rates of 626 and 577 µmol g^−1^ h^−1^, respectively, which are 1.4 and 1.2 times higher compared to the pristine case. Notably, the HER rate observed in this study (564 µmol g^−1^ h^−1^) was somewhat lower than that reported in previous studies on F8BT, possibly owing to the presence of relatively low residual palladium content within F8BT nanoparticles (16 ppm, measured by inductively coupled plasma mass spectroscopy, ICP‐MS).^[^ ^34^ ^]^ Furthermore, since the equivalent residual Pd in F8BT is the same as in each photocrosslinked photocatalysts, respectively, we speculated that the co‐catalytic effect of Pd would similarly influence the performance of each crosslinked F8BT nanoparticle. We also measured the performance of photocrosslinked F8BT nanoparticles over a wide range of optical wavelengths. Similar to the *λ* > 420 nm bandpass filter condition, the performance trend of each photocrosslinked photocatalysts were maintained with a light source with a *λ* > 350 nm bandpass filters (Table S1, Supporting Information). The remarkable HER enhancement observed with CL‐TEG crosslinking, compared to the lack of such an enhancement with hydrophobic crosslinkers such as CL‐C2 and CL‐C11, indicates that charges generated by the polymer semiconductor crosslinked with the hydrophilic crosslinker CL‐TEG react more strongly with the proton or hole scavenger. Drawing on prior studies, we surmise this effect parallels that observed with hydrophilic side chains, a topic to be elaborated in the subsequent section. Concerning the apparent quantum yield (AQY), the CL‐TEG‐crosslinked photocatalyst demonstrated a remarkable estimated efficiency of 0.3% at 420 nm and 0.4% at 450 nm, outperforming non‐crosslinked polymer photocatalysts. Furthermore, the photocatalytic stability of these photocrosslinked materials was assessed using CL‐TEG. After each 5 h reaction cycle, we employed high‐purity argon (>99.99%) to purge the reaction solution, a step primarily aimed at mitigating potential hole scavenger deficiencies. The reactivity of the photocrosslinked photocatalysts remained high (≈95%) until the completion of the first cycle. It is noted that the photocatalytic activity was efficiently restored by reintroducing fresh ascorbic acid, as particularly evident during the third cycle. This finding underscores the robust and recoverable characteristics of these photocrosslinked photocatalysts, holding their potential as sustainable candidates for catalytic applications.^[^ ^35^ ^]^ We also used UV–vis spectroscopy to confirm the stability of the photocrosslinked F8BT nanoparticles under photocatalytic conditions (Figure S17, Supporting Information). Comparing before and after photocatalytic reaction, we observed no change in the spectral properties of the entire photocrosslinked nanoparticle, indicating the stability of photocrosslinked F8BT under photocatalytic conditions.

### 2.4 Steady‐State Optical Properties

We analyzed the UV–vis absorption spectra of F8BT nanoparticles crosslinked with various agents (CL‐C2, CL‐C11, and CL‐TEG) dispersed in an aqueous medium. The absorption spectra of these photocrosslinked F8BT nanoparticles extended from 300 to 520 nm, prominently featuring the visible wavelength range, as depicted in Figure S13a (Supporting Information). The UV–vis spectra of the nanoparticles, both with and without crosslinking agents, show no significant differences. This indicates that the integration of crosslinking agents does not markedly alter the *π*‐electron conjugation of the F8BT polymer. In photoluminescence (PL) studies, the conjugated polymer nanoparticles with different crosslinkers displayed a primary peak at 543 nm when excited at 450 nm. A notably intense emission at a longer wavelength of 573 nm was observed for F8BT nanoparticles crosslinked with hydrophobic agents. This finding is consistent with a prior study suggesting that the PL spectra of such polymers exhibit distinct emission spectra in the long‐wavelength region, depending on the aggregation of aliphatic side chains (refer to Figures S18b and S19, Supporting Information).^[^ ^23^ ^]^ Interestingly, this enhanced aggregation did not significantly affect exciton or charge carrier dynamics, as will be elaborated on later. We found that the hydrophilicity of the crosslinker played a more critical role in HER enhancement, as discussed in the following sections.

### 2.5 Transient Optical Properties

**Figure 5:**
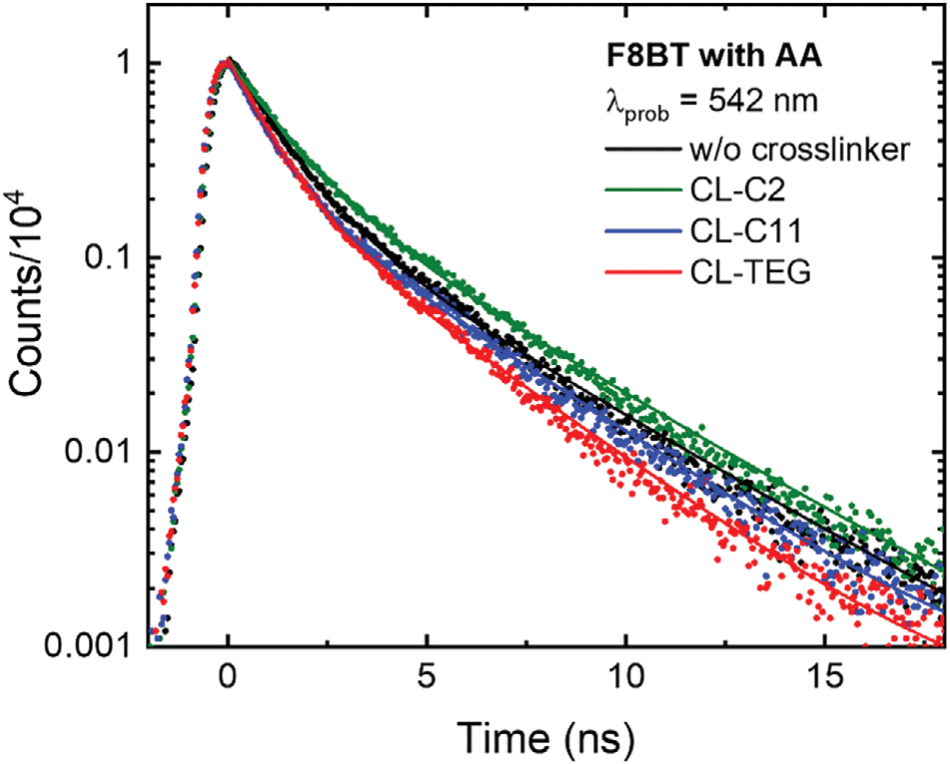
Time‐resolved photoluminescence (TR‐PL) decay kinetics of F8BT nanoparticles excited at 355 nm and probed at 542 nm, measured via time‐correlated single‐photon counting (TCSPC). The F8BT nanoparticles prepared with various photocrosslinkers were dispersed in H_2_O with ascorbic acid (pH 4, buffer NaOH). All nanoparticle solutions were prepared with an absorbance of 0.2 at the excitation wavelength.

For a more comprehensive analysis of charge carrier dynamics, we conducted time‐resolved photoluminescence (TR‐PL) decay kinetics studies on F8BT nanoparticles. These nanoparticles were excited at 355 nm and probed at 532 nm using time‐correlated single‐photon counting (TCSPC). Generally, the short decays of PL indicate promoted exciton recombination in photocatalysts. However, the decrease in lifetime with and without hole scavengers implies that the photogenerated excitons of the photocatalytic nanoparticles are immediately transferred to the hole scavenger and residual Pd.^[^ ^37^ ^]^ No significant differences in TR‐PL were discernible in scenarios lacking hole scavengers, as illustrated in Figure S20 (Supporting Information). However, in the presence of hole scavengers, F8BT nanoparticles that underwent photocrosslinking with CL‐TEG exhibited significantly shortened PL lifetimes, as shown in **Figure** **5**. This observation is attributed to the more effective exciton quenching from F8BT to the hole scavenger and proton, contrasting both non‐photocrosslinked nanoparticles and those treated with other crosslinkers, CL‐C2 and CL‐C11. The influence of hole scavengers on PL decay kinetics was unclear, potentially owing to the residual metal co‐catalysts in F8BT, which complicate the observation of the decay of photo‐induced excitons. However, in the presence of ascorbic acid, the effect of hydrophilic photocrosslinking agents on PL decay became more evident. This PL decay suggests potentially enhanced electron diffusion to the residual palladium particularly in hydrophilic photocrosslinker‐based nanoparticles. The time constants derived from bi‐exponential fits of the TR‐PL data are compiled in Table S2 (Supporting Information). This analysis discloses the effect of photocrosslinking agents on the photophysical properties of F8BT nanoparticles, in the context of enhancing HER performance.

**Figure 6:**
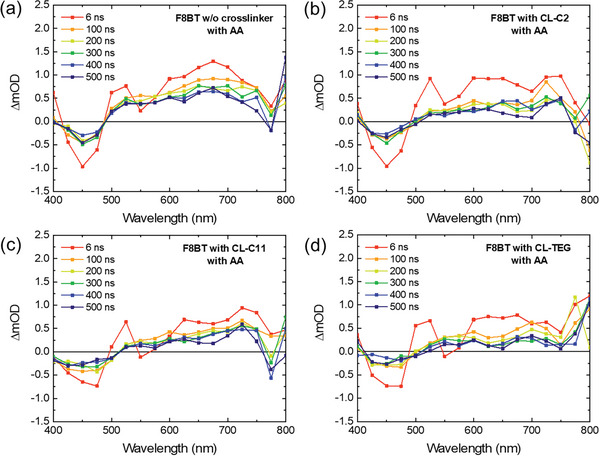
Transient absorption spectra of F8BT nanoparticles a) without crosslinker and photocrosslinked with b) CL‐C2, c) CL‐C11, and d) CL‐TEG in the presence of ascorbic acid after photoexcitation with a pump wavelength of 355 nm (2 mJ cm^−2^).

The analysis and comparison of photogenerated charge carrier kinetics in the polymer photocatalysts were supplemented by transient absorption (TA) spectroscopy. This method enables the investigation of the photophysical processes related to the presence and types of crosslinkers. We focused on the nanosecond timescale post‐photoexcitation, examining the reaction intermediate characteristics within the range of 400–800 nm. The effect of various crosslinkers on these characteristics was assessed by observing charge behaviors within the timescale of 8–500 ns following laser excitation at 355 and 532 nm, as shown in **Figure** **6**. The TA kinetic analysis, presented in Figure S21 (Supporting Information), shows that the F8BT nanoparticles without photocrosslinking exhibit a shorter charge carrier lifespan. In contrast, F8BT nanoparticles treated with the CL‐TEG crosslinker agent display an extended carrier lifetime, reaching tens of nanoseconds. This suggests a more effective conversion of excitons into free charges when hydrophilic crosslinkers are present.^[^ ^37^ ^]^ When introducing a hole scavenger, the TA spectra represent ground‐state bleaching (GSB) between 400 and 500 nm and excited‐state absorption (ESA) from 500 to 800 nm, as shown in Figure 6. The peaks of GSB appeared due to the absorption and spontaneous emission of F8BT nanoparticles, in addition, the peaks of ESA were depicted resulting from photo‐generated polarons. Compared to the non‐photocrosslinked F8BT nanoparticles, those treated with a hydrophilic crosslinker (CL‐TEG) exhibit a more rapid charge transfer, especially in the ESA region. To gain a deeper understanding of the kinetics over extended timescales, we analyzed the TA kinetics of the ESA region, ranging from nanoseconds to microseconds, in the presence of a hole scavenger for F8BT nanoparticles, as depicted in Figure S21b and Tables S3 and S4 (Supporting Information). The crosslinked F8BT nanoparticles showed the enhanced charge transfer rates particularly in the ESA feature, indicating suppressed charge accumulation during operation. This is likely attributable to the operational stability of F8BT nanoparticles photocrosslinked with CL‐TEG. Conversely, the non‐photocrosslinked F8BT nanoparticles display a longer carrier lifetime, suggesting the accumulation of photogenerated charges. Additionally, regarding the TA kinetics at the GSB, F8BT nanoparticles with CL‐TEG demonstrate a marginally quicker decay than that of their non‐crosslinked counterparts. This observation is consistent with the exciton transfer results from our TR‐PL analysis.

### 2.6 Water Contact Angle

The glycol side chains in polymer photocatalysts influence several aspects, such as the accessibility of proton/water and hole/electron scavengers to the polymer backbones and the charge carrier dynamics associated with excitons and long‐lived carriers. We performed static contact angle measurements to examine the effect of each crosslinker on the surface energies of the polymer photocatalysts (Figure S22, Supporting Information). The contact angles and surface energies of the photocrosslinked polymers are summarized in Table S5 (Supporting Information). The contact angle measurements reveal that the tetraethylene glycol chain in CL‐TEG confers the most pronounced hydrophilic properties to the conjugated polymers, surpassing other crosslinkers in reducing the surface energy of F8BT. As further discussed below, in molecular dynamics simulations, hydrophilic crosslinkers facilitate the hydration of conjugated polymers by enhancing water affinity and reducing surface energy.

### 2.7 Molecular Dynamics (MD) Simulation

To elucidate the functional role of CLs in enhancing the efficacy of polymer photocatalysts, we conducted extensive MD simulations. Initially, each of CL‐C11 and CL‐TEG were mixed with F8BT polymers and equilibrated in water. The F8BT polymer photocatalysts and photocrosslinkers were modeled using the OPLS‐AA force field (FF) with all‐atomistic representations.^[^ ^38^ ^]^ We determined the point charges of the CLs and the torsional parameters at the junctions between the F8‐BT monomers in the polymer backbone in this study. All reference FFs and the newly developed FF parameters are summarized in Figures S23 and S24 and Tables S6–S8 (Supporting Information). After initial equilibration, we retained the water molecules within the first solvation shell of the CLs to simulate the migration of the polymer/CL mixture into the polymer matrix. Further equilibration and computational crosslinking were carried out, as detailed in Section S11 (Supporting Information). As illustrated in Figure S25 (Supporting Information), computational crosslinking occurred between the photogenerated carbene sites of the crosslinkers and the nearest carbon atoms of the F8 alkyl side chains in the equilibrated polymer/CL mixture. For the polymer/CL mixtures with both CL‐TEG and CL‐C11, all alkyl carbon positions from the root to the end of the side chain participated in photocrosslinking, with the chain end showing a higher, but not exclusive, selectivity (Figure S26, Supporting Information). This indicates that the crosslinkers can bridge positions close to the F8BT polymer backbones, and introducing hydrophilic crosslinkers effectively enhances the water affinity of these backbones.

**Figure 7:**
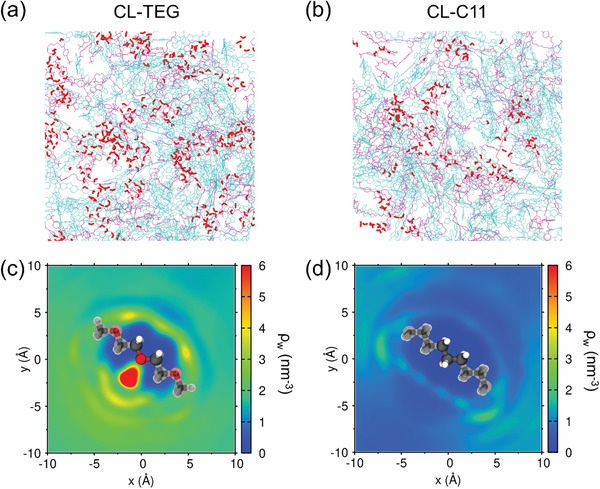
Water coordination structures on the crosslinkers predicted via molecular dynamics simulations. Snapshots of F8BT matrices with a) CL‐TEG and b) CL‐C11, coexisting with a 33.3 wt.% water content. Water molecules within 3.5 Å of the TEG or C11 bridges are highlighted in red, and the bridges and F8BT chains are colored in magenta and cyan, respectively. Spatial distribution functions of water molecules around c) ethylene oxide moieties of CL‐TEG and d) propane moieties of CL‐C11 bridges projected on the molecular plane. ρ_w_ notates the average number density of water molecules within 3.0 Å above and below the molecular plane. The reference molecules are drawn with their actual sizes and orientations.

As demonstrated by the equilibrated structures of the photocrosslinked polymers with CL‐TEG and CL‐C11 (**Figure** **7**a,b), the hydrophilic CL‐TEG notably increases the number of water molecules solvating the linker compared to CL‐C11. This increase in hydrating water molecules around the CL‐TEG can be maintained when incorporated into the polymer matrix, improving water permeation. This observation aligns with the experimental contact angle measurements (Figure S22, Supporting Information). The increased hydration of the linker is quantifiable via the radial (Figure S28, Supporting Information) and spatial (Figure 7c,d) distribution functions (RDFs and SDFs) of water molecules around the linkers. Here, CL‐TEG exhibits a characteristic peak indicating strong hydrogen bonding approximately at 2.8 Å from the oxygen atom and a consistently higher number of water molecules within up to 1 nm distance compared to the C11 linker. The SDF of water near the TEG linker also accentuates the accumulation of water molecules near the TEG oxygen, forming a robust hydrogen bond (vivid red area in Figure 7c). This extends to multiple layers of solvated water molecules around the CL‐TEG (Figure S29, Supporting Information). In contrast, CL‐C11 shows no significant aggregation of adjacent water molecules (Figure S28, Supporting Information). The water distribution patterns after exciton generation suggest that linker‐mediated water penetration is crucial for HER and the stabilization of charge carriers.

**Figure 8:**
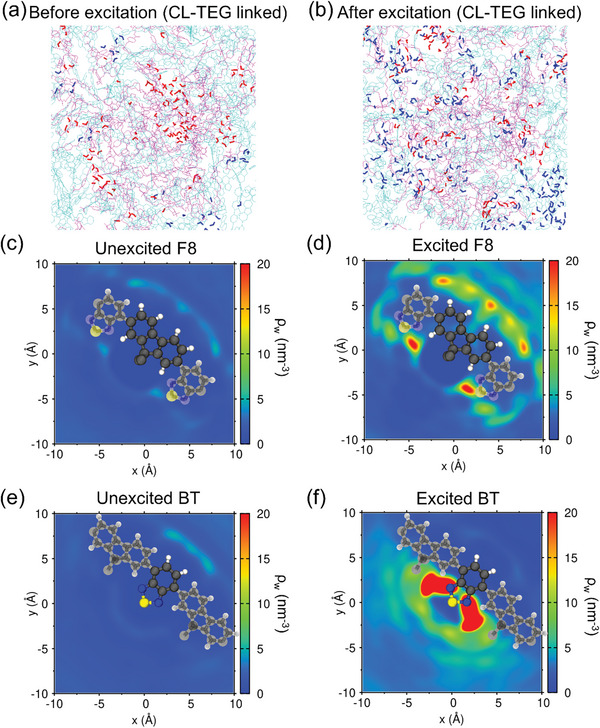
Water coordination structures on F8BT before and after exciton generation predicted in molecular dynamics simulations of the F8BT photocrosslinked via CL‐TEG. Snapshots a) before and b) after exciton generation on randomly chosen 20% of F8BT copolymer units. Crosslinkers and F8BT chains are colored with magenta and cyan, respectively. Water molecules hydrating the TEG bridges and exciton‐containing F8BT monomers within a 3.5 Å distance are highlighted with red and blue, respectively. SDFs of water molecules around the conjugated parts of c) unexcited F8 units, d) excited F8 units, e) unexcited BT units, and f) excited BT units projected on the molecular plane. ρ_w_ notates the average number density of water molecules within 3.0 Å above and below the molecular plane. The reference molecules are drawn with their actual sizes and orientations. The alkyl side chains of the F8 unit are omitted for clarity.

The functional role of linker‐mediated water permeation in stabilizing charge carriers against undesirable recombination is an important aspect to be considered. We developed a computational model simulating exciton in the F8BT copolymer unit (Figure S27, Supporting Information). In the photocrosslinked polymer matrix with CL‐TEG, we randomly selected 20% of the F8BT copolymer units to generate charge carriers (see Section S12, Supporting Information for detailed methodology). The hydrating water molecules located near the TEG bridges before excitation (**Figure** **8**a) were observed to migrate toward the vicinity of the excited F8BT copolymer units (Figure 8b). Both the RDFs (Figure S30, Supporting Information) and SDFs (Figure 8c–f) of water molecules around the conjugated atoms of the F8 and BT units reveal that post‐excitation, the hydration of the F8BT units becomes significantly favorable, in contrast to their minimal hydration shells in unexcited states. In areas densely hydrated around the excited F8BT units (Figure 8f), the local water molecule density surpasses that of bulk water under ambient conditions (≈33.4 nm^−3^), highlighting the extent of linker‐mediated penetration of water molecule to hydrate the excitons resulting in stabilization of charge carriers. Notably, the hydration of the excited BT units predominantly occurs around the nitrogen atoms. This is attributed to the intensified negative partial charge distribution upon exciton generation, leading to strong interactions with the hydrogen atoms of water (Table S9, Supporting Information). In a previous experimental study, the nitrogen atoms in the BT anion radical were posited as active sites for catalytic hydrogen production via proton acceptance.^[^ ^39^, ^40^ ^]^ Intriguingly, these specific interatomic interactions highlighted in our simulation prediction align with the reaction preferences reported in experimental studies.

### 2.8 Universal Application of CL‐TEG to Other Polymer Photocatalysts

To validate the universality of our proposed photocrosslinking approach across common conjugated polymers, we assessed the HER performance of PFODTBT and P(BTC8‐T). These representative conjugated polymers, incorporating benzothiadiazole (BT) units known for their photocatalytic properties, are illustrated in Figure S31 (Supporting Information).^[^ ^39^ ^]^ Remarkably, all photocrosslinked polymer photocatalysts utilizing CL‐TEG exhibit superior performance, achieving HER ratios more than twice as high as their non‐crosslinked counterparts. This demonstrates the versatility of hydrophilic crosslinkers for various polymer photocatalysts beyond F8BT. As discussed earlier, this enhancement is ascribed to the facilitation of improved water access to the polymer by the photocrosslinking agent, stabilization of charge carriers generated via photoexcitation, and extended lifetimes of the separated exciton charge carriers. Thus, our strategy employing hydrophilic photocrosslinkers hold a promising potential for universal application as evidence by the increase in the HER performance of other conjugated polymer photocatalysts.

## 3 Conclusion

In this study, we comparatively assessed the impact of photocrosslinker properties on the efficacy of polymer photocatalysts. A significant enhancement in performance was observed when using a hydrophilic photocrosslinking agent, achieved via careful modification of their chemical structures. FT‐IR analysis provided conclusive evidence of a successful reaction between the bisdiazirine photocrosslinkers and alkyl side chains of F8BT within the nanoparticles while maintaining similar particle sizes as evidenced by DLS and LP‐TEM measurements. The hydrophilic photocrosslinker significantly affected the internal conditions of the nanoparticles, resulting in notable changes in photophysics and molecular dynamics. TR‐PL measurements indicated a faster decay of excitons under hydrophilic conditions than under hydrophobic conditions. Moreover, we observed the prolonged lifetime of long‐lived charge carriers in the absence of hole scavengers. Their transport to the co‐catalysts was facilitated in the presence of hole scavengers, as supported by TA measurements within the nanosecond‐to‐microsecond range. These findings suggest that the hydrophilic interior conditions of the nanoparticles extend the lifetimes of charge carriers and enhance proton reduction. Contact angle measurements and structural in‐depth study using MD simulations showed that the hydrophilic photocrosslinker significantly enhanced water penetration into the polymer matrix. Such linker‐mediated water penetration is crucial for hydrating excitons and stabilizing charge carriers. Consequently, the hydrophilic photocrosslinking agent resulted in a notable 2.5‐fold increase in the performance of polymer catalysts compared to those without photocrosslinking. This study underscores the potential of hydrophilic crosslinking as a viable approach for enhancing hydrogen production efficiency in photocatalysts.

## Conflict of Interest

The authors declare no conflict of interest.

## Supporting information

Supporting Information

## Data Availability

The data that support the findings of this study are available from the corresponding author upon reasonable request.
