# Supplementary material for: Hydrophilic Photocrosslinkers as a Universal Solution to Endow Water Affinity to a Polymer Photocatalyst for an Enhanced Hydrogen Evolution Rate
Source: Adv Sci (Weinh). 2024 May 17;11(28):2309786. doi: 10.1002/advs.202309786 (PMC11267343; doi:10.1002/advs.202309786)
Supplement: Supplementary file 1 — Supporting Information [file ADVS-11-2309786-s001.docx]

**Supporting information**

**Experimental**

*1. Material Synthesis*

*1.1 Synthetic scheme of photocrosslinkers*

Figure S1. Synthetic scheme of CL-C2, CL-C11, and CL-TEG.

*1.2 General Synthetic Procedures*

All reagents were obtained from commercial suppliers and used without further purification. N,N'-dicyclohexylcarbodiimide (DCC) (1.0 M in methylene chloride), anhydrous dichloromethane (DCM), and CDCl_3_ were purchased from Sigma Aldrich. (1) 4-[3-(trifluoromethyl)-3 H -diazirin-3-yl]benzoic acid were synthesized following the earlier report.^[S1]^ 4-(Dimethylamino)pyridine (DMAP), (2) ethylene glycol, (3) 1,11-undecane-diol, (4) tetraethylene glycol were purchased from TCI Chemicals. MgSO_4_, silica gel, dichloromethane, n-hexane and ethyl acetate were purchased from Daejung Chemicals Korea. F8BT, PFODTBT, and SDS were purchased from Sigma Aldrich. P(BTC8-T) was synthesized according to previously reported methods.^[S2] 1^H NMR, ^13^C NMR and ^19^F NMR spectra were recorded with a Bruker ADVANCE-400 spectrometer which operated at 400 MHz for ^1^H nuclei, 100 MHz for ^13^C nuclei, and 376.5 MHz for ^19^F nuclei and are internally referenced to residual solvent signals. Chemical shifts are reported in parts per million (ppm).

*1.3 Synthesis of Photocrosslinkers*

**CL-C2:**

A mixture of (1) 4-[3-(trifluoromethyl)-3 H -diazirin-3-yl]benzoic acid (200 mg, 0.87 mmol) and (2) ethylene glycol (21 µL, 0.39 mmol) were dissolved in anhydrous dichloromethane (25 mL) under stirring at room temperature followed by addition of DMAP (21mg , 0.17 mmol). After 30 minutes, the temperature was lowered to 0 ºC, and DCC (1 M in dichloromethane) (0.95 mL, 0.95 mmol) was added under N_2_ atmosphere. After 12 h, the reaction mixture was neutralized with water and extracted with dichloromethane and solvents were removed by rotary evaporator at a reduced pressure. The resulting crude product was purified by silica gel column chromatography using an eluent of ethyl acetate/n-hexane (1/5), which yielded the CL-C2 as a colorless solid (132 mg, 60 %).

^1^H NMR (400 MHz, CDCl_3_): δ = 4.69 (m, 4H), 7.27 (d, 4H), 8.08 (d, 4H),

^13^C NMR (100 MHz, CDCl_3_): δ = 62.95, 120.47, 123.20, 126.44, 130.01, 130.77, 134.09, 165.25.

^19^F NMR (376.5 MHz, CDCl_3_): δ = 64.94.

**CL-C11:**

A mixture of (1) 4-[3-(trifluoromethyl)-3 H -diazirin-3-yl]benzoic acid (200 mg, 0.87 mmol) and (3) 1,11-undecane-diol (73 mg, 0.39 mmol) were dissolved in anhydrous dichloromethane (25 mL) under stirring at room temperature followed by addition of DMAP (21mg , 0.17 mmol). After 30 minutes, the temperature was lowered to 0 ºC, and DCC (1 M in dichloromethane) (0.95 mL, 0.95 mmol) was added under N_2_ atmosphere. After 12 h, the reaction mixture was neutralized with water and extracted with dichloromethane and solvents were removed by rotary evaporator at a reduced pressure. The resulting crude product was purified by silica gel column chromatography using an eluent of ethyl acetate/n-hexane (1/10), which yielded the CL-C11 as a yellow liquid (165 mg, 62 %).

^1^H NMR (400 MHz, CDCl_3_): δ = 1.32-1.44 (br, 14H), 1.76 (m, 4H), 4.34 (t, 4H), 7.27 (d, 4H), 8.07 (d, 4H).

^13^C NMR (100 MHz, CDCl_3_): δ = 25.98, 28.64, 29.22, 29.46, 65.51, 120.51, 123.24, 126.31, 129.86, 131.56, 133.60, 133.61, 165.57.

^19^F NMR (376.5 MHz, CDCl_3_): δ = -64.96.

**CL-TEG:**

A mixture of (1) 4-[3-(trifluoromethyl)-3 H -diazirin-3-yl]benzoic acid (200 mg, 0.87 mmol) and (4) tetraethylene glycol (67 µL, 0.39 mmol) were dissolved in anhydrous dichloromethane (25 mL) under stirring at room temperature followed by addition of DMAP (21mg , 0.17 mmol). After 30 minutes, the temperature was lowered to 0 ºC, and DCC (1 M in dichloromethane) (0.95 mL, 0.95 mmol) was added under N_2_ atmosphere. After 12 h, the reaction mixture was neutralized with water and extracted with dichloromethane and solvents were removed by rotary evaporator at a reduced pressure. The resulting crude product was purified by silica gel column chromatography using an eluent of ethyl acetate/n-hexane (1/2), which yielded the CL-TEG as a yellow liquid (147 mg, 52 %).

^1^H NMR (400 MHz, CDCl_3_): δ = 3.68 (br, 8H), 3.83 (t, 4H), 7.25 (d, 4H), 8.08 (d, 4H).

^13^C NMR (100 MHz, CDCl_3_): δ = 64.41, 69.10, 70.67, 120.48, 123.21, 126.31, 130.00, 131.19, 133.79, 165.44.

^19^F NMR (376.5 MHz, CDCl_3_): δ = 64.96.

**Carbene Insertion Test**

To investigate the C-H insertion reaction, we prepared two samples by dissolving CL-C11 (5 mg) into cyclohexane (4 mL). One sample underwent drying without UV irradiation, while the other was exposed to UV light for 30 minutes under stirring conditions to enhance carbene generation for subsequent C-H insertion. After evaporating excess cyclohexane, the samples were dissolved in CDCl_3_ and characterized using ^1^H NMR and ^19^F NMR. It is important to note that excess cyclohexane was evaporated to mitigate errors during NMR characterization.

*1.4 Characterization of Photocrosslinkers*

**Characterization of CL-C2**

**^1^H NMR**

Figure S2. ^1^H-NMR spectrum of CL-C2 in CDCl_3_.

**^13^C NMR**

Figure S3. ^13^C-NMR spectrum of CL-C2 in CDCl_3_.

**^19^F NMR**

Figure S4. ^19^F-NMR spectrum of CL-C2 in CDCl_3_.

**Characterization of CL-C11**

**^1^H NMR**

Figure S5. ^1^H-NMR spectrum of CL-C11 in CDCl_3_.

**^13^C NMR**

Figure S6. ^13^C-NMR spectrum of CL-C11 in CDCl_3_.

**^19^F NMR**

Figure S7. ^19^F-NMR spectrum of CL-C11 in CDCl_3_.

**Characterization of CL-TEG**

**^1^H NMR**

Figure S8. ^13^C-NMR spectrum of CL-TEG in CDCl_3_.

**^13^C NMR**

Figure S9. ^1^H-NMR spectrum of CL-TEG in CDCl_3_.

**^19^F NMR**

Figure S10. ^19^F-NMR spectrum of CL-TEG in CDCl_3_.

*2. Nanoparticle Synthesis via Mini-emulsion*

Polymer stock solutions were prepared in chloroform (5 mg/mL) and photocrosslinkers were dissolved in chloroform (1 mg/mL). The solutions were heated for 1 h at 40 °C to ensure complete dissolution. To match the molar ratio of photocrosslinkers to the mass of the conjugated polymer, conjugated polymer (1 mg) and photocrosslinkers (CL-C2: 0.09 mg, CL-C11: 0.24 mg, CL-TEG: 0.3 mg) were dissolved in 1 mL chloroform. For the precursor solution in chloroform was added to SDS / deionized water (50 mg /10 mL) in the aqueous phase. The solution with chloroform and water was ultrasonicated with an ultrasonic processor (Sonics VibracCell VCX-750, 750 W 30% amplitude) for approximately 5 mins to make a mini-emulsion where the oil phase was dispersed in the aqueous phase. The mini-emulsion solution was heated at 85 °C on a hot plate to evaporate the chloroform. To remove large aggregations, the dispersion was filtered with a 0.4 μm PVDF syringe filter.

*3.UV-Visible Absorption Spectroscopy*

A Thermo Scientific Evolution 220 UV–visible absorption spectrophotometer was used to obtain the UV-visible absorption spectra.

*4. Dynamic Light Scattering (DLS), and Contact Angle*

The size distribution of each nanoparticle batch was measured with a Malvern Zetasizer Nano S (Malvern Instruments Nordic AB). Average data were obtained from at least five runs of measurements. The contact angle goniometer and software were provided by Ossila, the average contact angle value was measured using droplet fitting after edge detection.

*5. Liquid Phase Transmission Electron Microscopy (LP-TEM)*

The size and morphology of conjugated polymer nanoparticles were analyzed by LP-TEM using graphene liquid cells. The graphene liquid cells were prepared by following a previously reported method.^S3^ The graphene-coated TEM grids were prepared through the direct transfer of graphene on Cu foils.^S4^ A tiny volume of the solution containing the conjugated polymer nanoparticles was encapsulated between two TEM grids coated with graphene. The sandwiched grids were clamped with tweezers and the excess solution was removed, forming graphene liquid cells. LP-TEM imaging was conducted using the Tecnai G2 F20 TWIN TMP (FEI, Thermo Fisher Scientific) operating at 200 kV.

*6. Hydrogen Evolution Rate (HER)*

Measurements were conducted in a 50 mL quartz flask. Conjugated polymer photocatalysts nanoparticles were dispersed into ascorbic acid solutions (0.2 M Ascorbic Acid pH 4, buffer with NaOH) (50 mL). After purging with Ar for 20 min to remove O_2_, the reaction mixture was illuminated with a 300-W Newport Xe light-source (Model: 66160, Ozone free) using a 420-nm cutoff filter. Hydrogen was detected with a thermal conductivity detector by comparison with a standard gas with a known concentration of hydrogen and analyzed on a Nexis GC-2030 gas chromatograph. Any hydrogen dissolved in the reaction mixture was not measured, and the pressure increase generated by the evolved hydrogen was neglected in the calculations. We used about 0.95 mg for the photocatalytic activity. The performance of the photocatalysts and its concentration are proportional up to a certain threshold, beyond which it reaches a satrurated state due to reflection, scattering and so on.^[S4]^ For these reasons, we use this concentration for higher absorbance under photocatalytic conditions.

*7. Apparent Quantum Yield (AQY)*

Apparent quantum yield (AQY) was determined under the conditions used for photocatalytic hydrogen generation experiments in a 3.5 mL airtight quartz cuvette (path length 1 cm). The solution was illuminated by a 300-W Newport Xe light-source (Model: 66160, Ozone free) as a light source e quipped with an 420 nm cutoff filter and monochromated with a bandpass filter to 420, 450, 500, and 550 nm. And AQY were calculated from following equation:

|  | $AQY\left( \% \right)=2\times\frac{moles of hydrogen}{moles of incident lights}=2\times\frac{M\times N_{A}\times h\times c}{A\times P\times t\times\lambda}\times100\%$*,* | (1) |
| --- | --- | --- |

where *M* is the production of H_2_ (mol sec^−1^); *N_A_* is Avogadro’s constant 6.02×10^23^ mol^−1^; *h* is Planck’s constant 6.62×10^−34^ J∙s; *c* is the light speed under vacuum 3.00×10^8^ m s^−1^; *A* is the area of light irradiation; *λ* is the light wavelength; *P* is the incident monochromatic light intensity at *λ*; and *t* is the irradiation time at *λ*.

*8. Time-resolved Photoluminescence (TR-PL)*

The TR-PL data were measured by the time-correlated single-photon-counting (TCSPC) method (Edinburgh Instruments Ltd, FS5 Spectrofluorometer) with an excitation wavelength of 540 nm.

*9. ns-μs Transient Absorption Spectroscopy(ns-TAS)*

Nanosecond – microsecond TA data were collected using a pump – probe TA spectroscopy, which consisted of a TA spectrometer and a regenerative amplified Nd:YAG laser (EL-YAG) with a pulse width of 6-8 ns. The pulse is capable of generating both visible pulses (532 nm) and UV pulses (355 nm) through a third harmonic generator. The TA spectra data were collected over a time range from 6 ns to 500 ns. The probe beam is derived from a 150 W Xenon lamp, which is reflected off the nanoparticle sample and then passed through a monochromator before reaching a PMT-980 photodiode detector. To simultaneously capture data on two different time scales, the comprehensive L900 spectrometer software (V9.4.3) package is utilized, and the nanosecond-microsecond signal is sampled using an oscilloscope (Tektronix MDO30232, Beaverton, OR, USA). Excitation fluences are measured using a pyroelectric energy senor.

*10. Fourier Transform Infrared spectroscopy (FT-IR)*

The FT-IR analysis was performed using a Fourier transform photocurrent analyzer (Vertex 79v, Bruker)

*11. Inductively Coupled Plasma-Mass Spectroscopy (ICP-MS)*

Metal contents were determined by inductively coupled plasma (ICP) spectroscopy on acid-digested F8BT samples. Pd contents were determined by ICP-MS (PerkinElmer ICP MS NexION 2000)

*12. Statistical Analysis*

The statistical analysis conducted in this study involved several steps, including data pre-processing, averaging, and calculating mean and standard deviation, which were performed using Origin 8 software. We conducted measurements on a group of 5 photocatalytic reactors to assess their hydrogen evolution performance parameters. Photocatalytic parameters were reported as mean ± standard deviation, offering a concise summary of the data from the 5 reactors. Our analysis, employing an independent samples t-test, revealed statistically significant enhancements in the photovoltaic parameters (P < 0.01). We utilized a combination of Origin 8 and Excel 2023 software for these statistical analyses. Furthermore, details of the molecular dynamics simulation methods were outlined in the Supporting Information. Computational predictions of radial distributions, cumulative coordination numbers, and spatial distributions were presented as means across simulation snapshots saved every 10 ps during a production run lasting 500 ns. Given that the standard deviation among time block averages of 100 ns was smaller than the line width of the figures, statistical error notations were omitted. The histogram depicting the carbon position of photocrosslinking to the F8 alkyl side chain was computed by averaging over simulation snapshots saved every 20 ps during NPT equilibration runs lasting 20 ns. Error bars indicate ± standard deviation across the recorded snapshots.


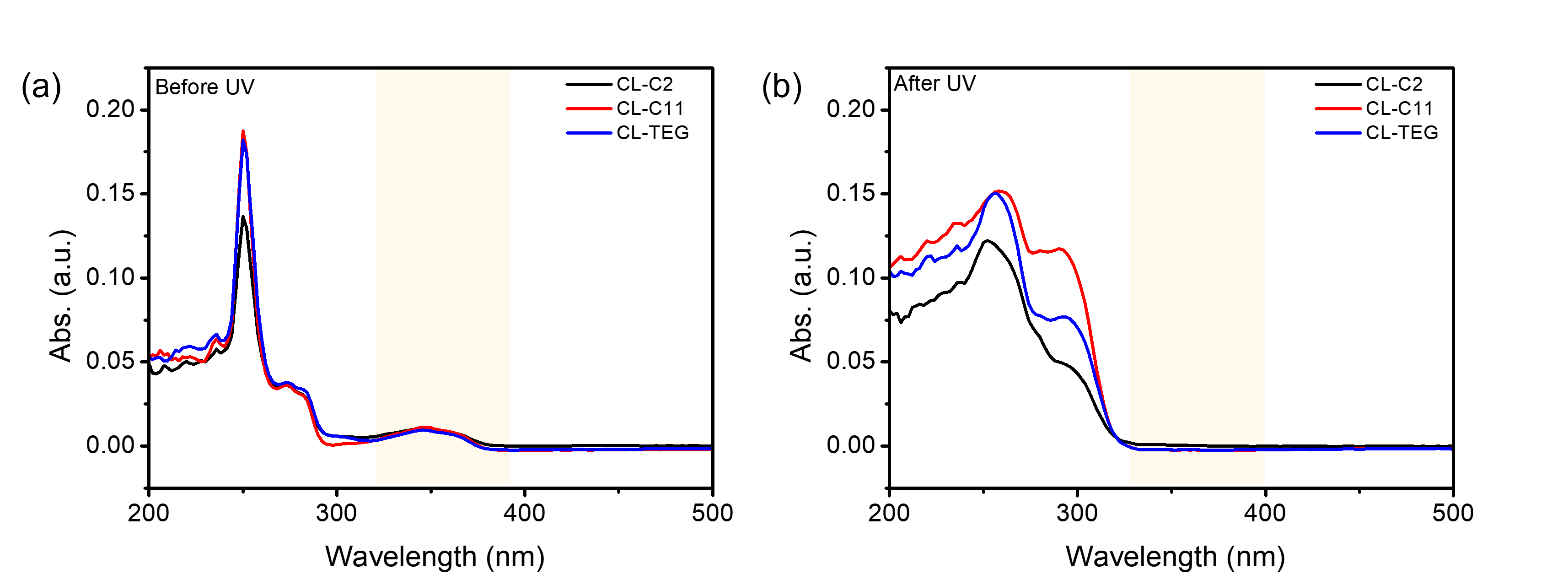


**Figure S11.** UV-Vis absorption spectra of photocrosslinkers in chloroform solutions (a) before and (b) after UV irradiation (365 nm), respectively. After UV irradiation, the nitrene absorption bands disappeared for each photocrosslinking agent.

**
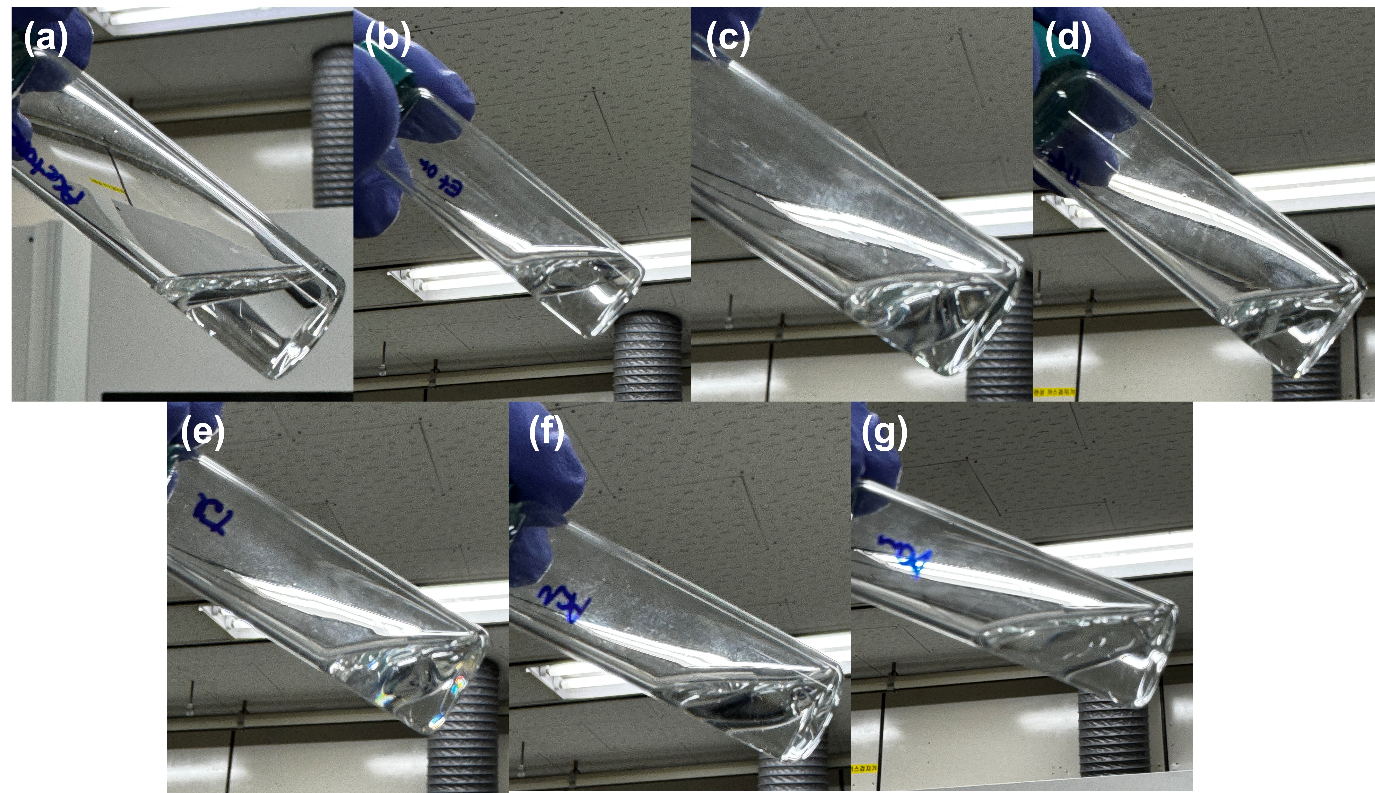
**

**Figure S12.** The images of solubility tests of CL-TEG in (a) acetone, (b) ethanol, (c) tetrahydrofuran, (d) toluene, (e) acetonitrile, and (g) dichloromethane, respectively


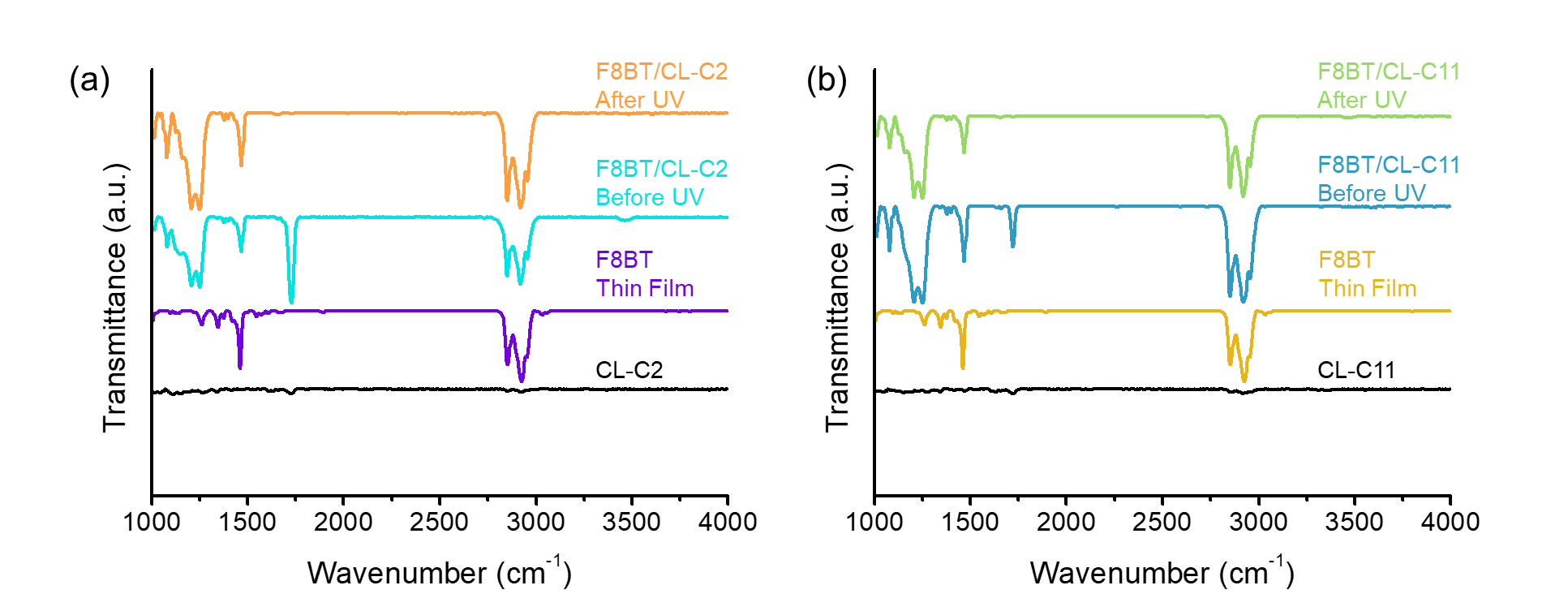


**Figure S13.** Solid-state FT-IR spectra of F8BT nanoparticles (a) CL-C2 and (b) CL-C11: (top) photocrosslinked F8BT nanoparticles with photocrosslinkers; (second) F8BT and photocrosslinkers nanoparticles without UV irradiation; (third) neat F8BT thin film; (bottom) neat photocrosslinkers thin film, respectively. After 365 nm UV light irradiation, diazirine band (1726 cm^−1^) was disappeared. All nanoparticles and thin films of F8BT and crosslinkers were drop-casted on Si wafers.


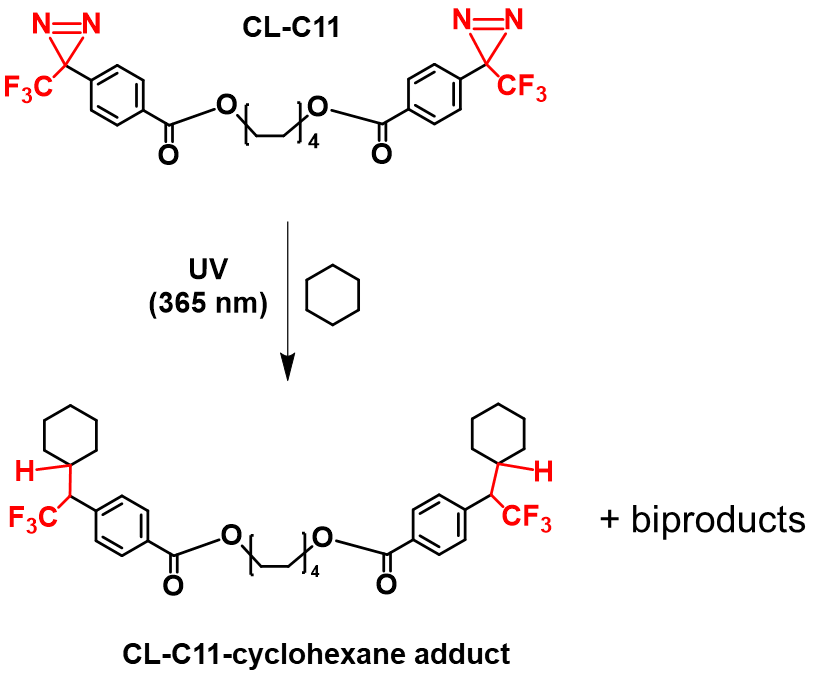


**Figure S14**. Illustration depicting the formation of the CL-C11-cyclohexane adduct through photochemically induced C-H insertion reaction.^[S5]^


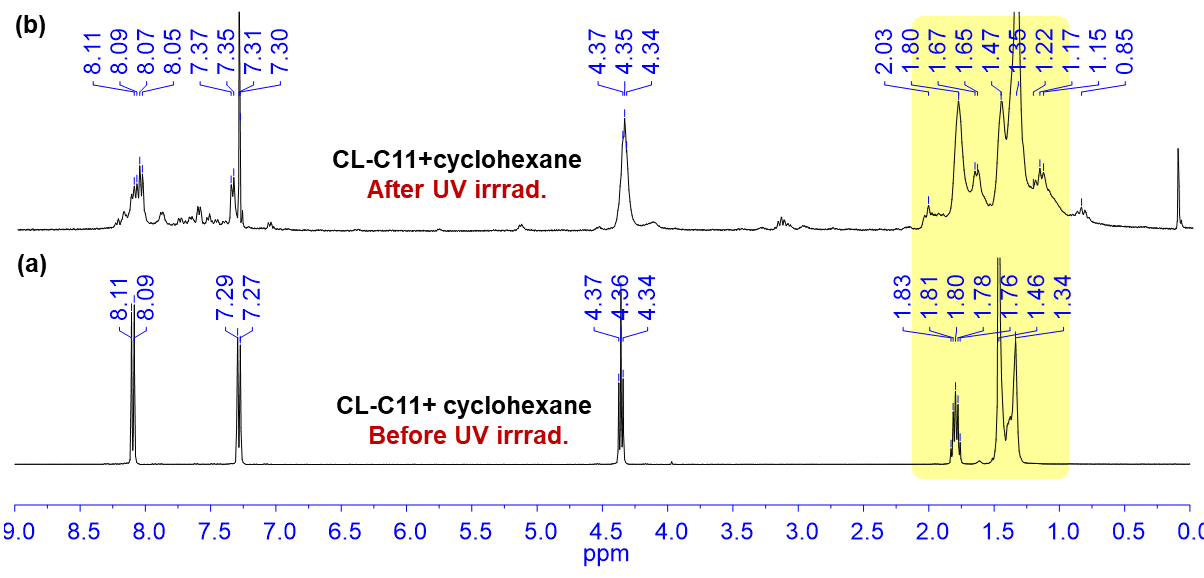


**Figure S15.** ^1^H NMR spectra of CL-C11 acquired before and after exposure to UV light (365 nm) irradiation.


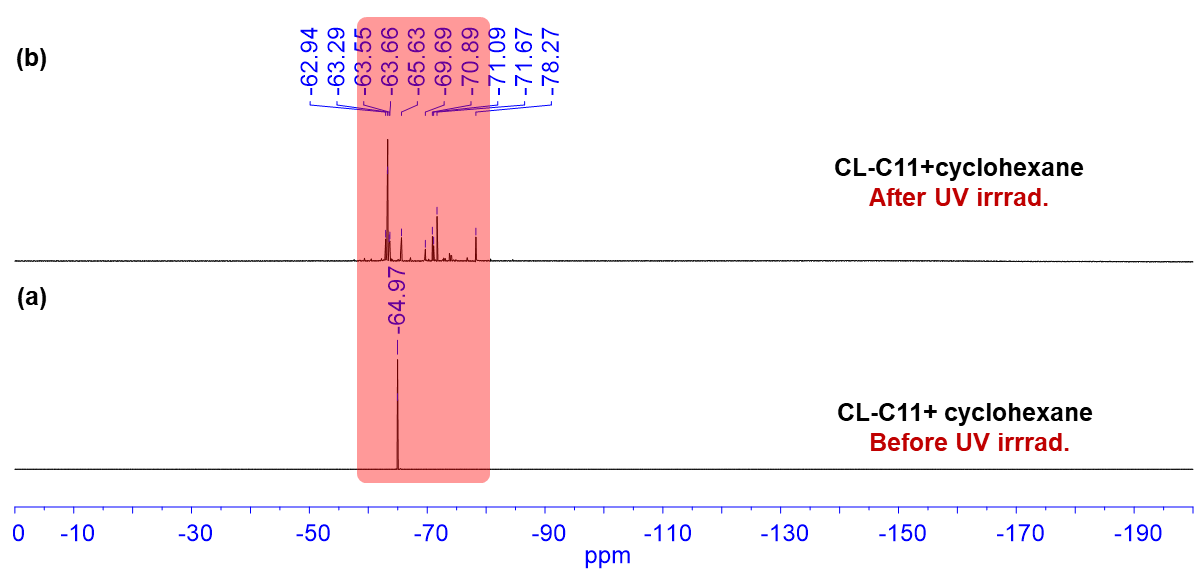


**Figure S16**. ^19^F NMR spectra of CL-C11 acquired before and after exposure to UV light (365 nm) irradiation.

Table S1. Summary of Hydrogen evolution performance for F8BT nanoparticle with light source with λ > 350 nm bandpass filters.

|  | HER (μmol g^−1^ h^−1^) |
| --- | --- |
| F8BT | 740 |
| F8BT:CL-C2 | 912 |
| F8BT:CL-C11 | 884 |
| F8BT:CL-TEG | 1760 |


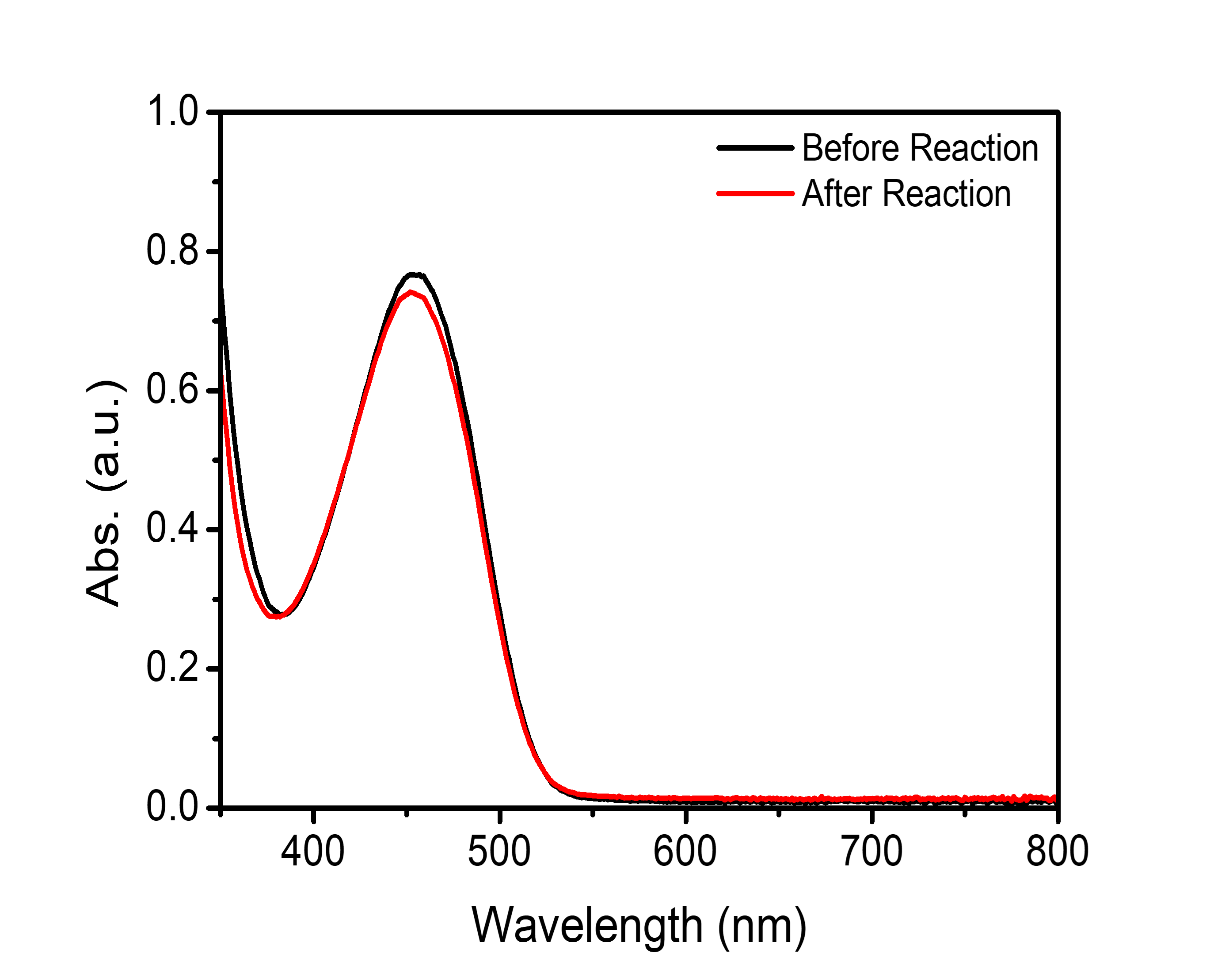


**Figure S17.** Absorption spectra of F8BT nanoparticles photocrosslinked with CL-TEG before (black) and after(red) photocatalysis.


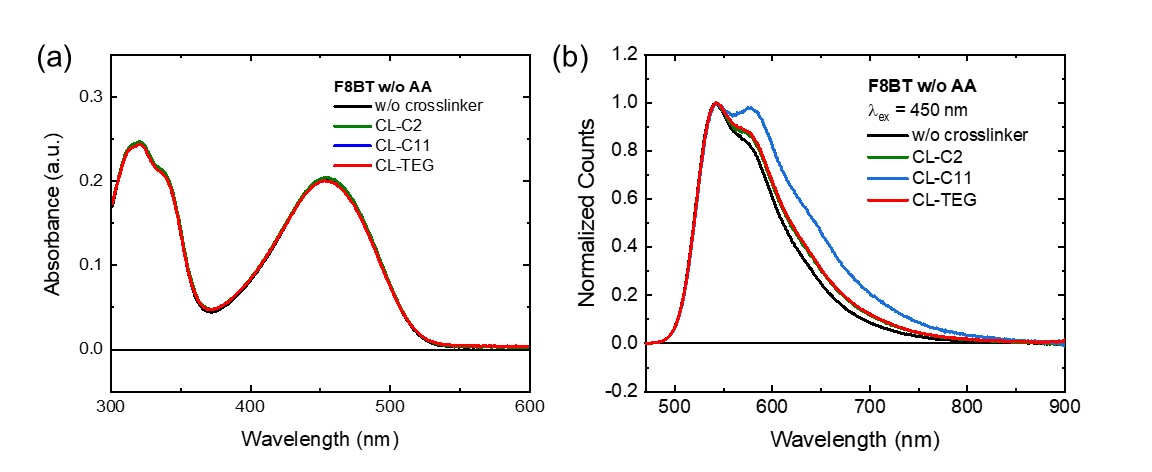


**Figure S18**. (a) UV-Vis absorption spectra and (b) PL spectra of F8BT nanoparticles without crosslinker, and photocrosslinked with CL-C2, CL-C11 and CL-TEG.


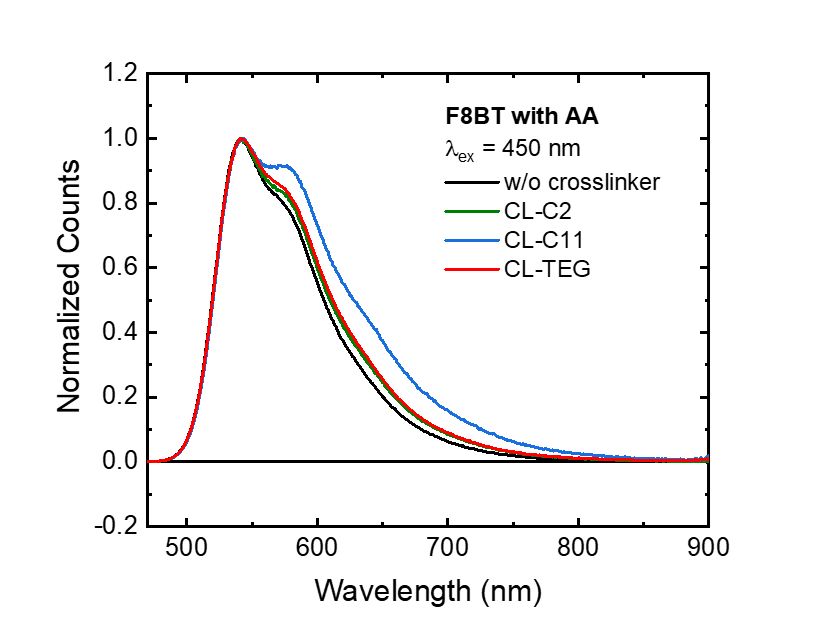


**Figure S19.** PL spectra of F8BT nanoparticles without crosslinker, and photocrosslinked with CL-C2, CL-C11 and CL-TEG in Ascorbic Acid (AA) solution (0.2 M, pH 4 buffer with NaOH), respectively.


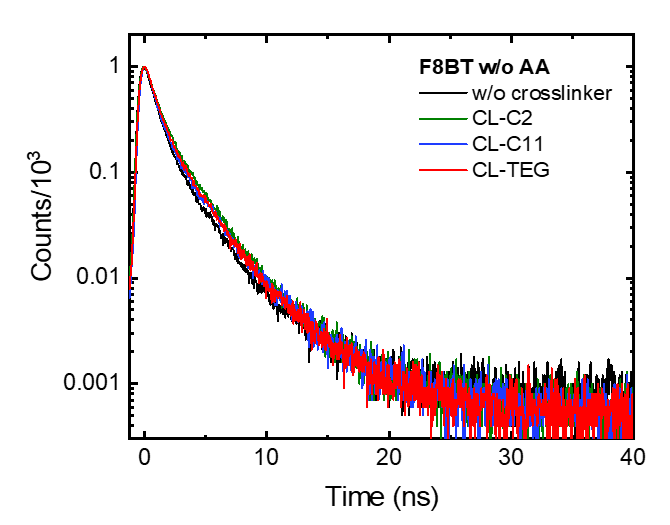


**Figure S20.** Time-resolved photoluminescence (TR-PL) decay kinetics of F8BT nanoparticles excited at 355 nm and probed at 542 nm, without hole scavenger condition.

**Table S2.** Kinetic parameters of PL decay photocrosslinked F8BT nanoparticles with various crosslinker agents.

|  | w/o crosslinker | CL-C2 | CL-C11 | CL-TEG |
| --- | --- | --- | --- | --- |
| τ_1_ (ns)^a)^ | 1.12 | 1.16 | 0.88 | 0.95 |
| A_1_ (%)^b)^ | 8790 | 7351 | 7537 | 7977 |
| τ_2_ (ns) | 3.72 | 3.53 | 3.22 | 3.02 |
| A_2_ (%) | 2318 | 3350 | 2774 | 2497 |
| τ_avg_ (ns)^c)^ | 1.66 | 1.90 | 1.50 | 1.22 |
| χ^2 d)^ | 1.3733 | 1.3882 | 1.3297 | 1.4326 |

1. kinetic parameters were obtained from stretched biexponential fits to the data as shown Figure 5, using $y= \int_{-\infty}^{t} IRT(t^{'})\sum A_{i}\exp\left( -\frac{t-t^{'}}{\tau_{i}} \right)dt^{'}$
2. fractional amplitudes of the positive decay components
3. average lifetimes of triexponential fits were obtained from amplitude weighted calculations
4. χ^2^ represents goodness-of-fit parameter and was obtained by using $\chi^{2}= \sum{[{(y}_{i}-f_{c})/(y_{i}^{2})]}^{2}$where *y_i_* is the measured data, *f*_c_ is the fitted data.


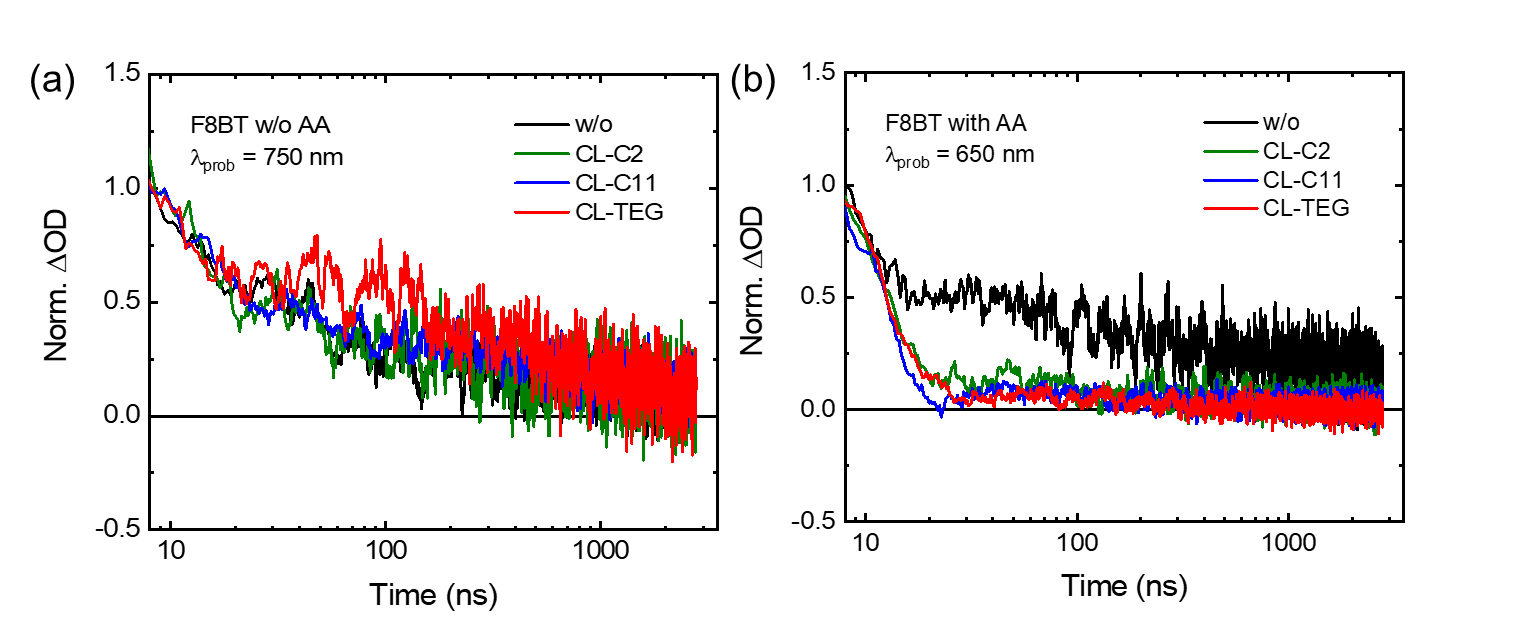


**Figure S21.** (a) Transient absorption decay kinetics of F8BT nanoparticles with photocrosslinkers monitored with a probe wavelength or 750 nm in the absence of AA. (b) Transient absorption decay kinetics of F8BT nanoparticles with photocrosslinkers monitored with a probe wavelength or 650 nm with AA. The transient absorption kinetics are monitored up to 3 μs after photoexcitation with a pump wavelength of 355 nm (2 mJ cm^−2^).

**Table S3.** Transient absorption decay kinetics parameters of photocrosslinked F8BT nanoparticles with various crosslinker agents probe at 750 nm in the absence of AA.

|  | A_1_ | τ_1_(ns) | A_2_ | τ_2_(ns) |
| --- | --- | --- | --- | --- |
| w/o crosslinker | 0.765 | 31.94 | 0.211 | 1436 |
| CL-C2 | 0.836 | 23.74 | 0.205 | 917.6 |
| CL-C11 | 1.027 | 14.52 | 0.253 | 746.4 |
| CL-TEG | 0.305 | 121.7 | 0.350 | 891.0 |

**Table S4** Transient absorption decay kinetics parameters of photocrosslinked F8BT nanoparticles with various crosslinker agents probe at 650 nm with AA.

|  | A_1_ | τ_1_(ns) | A_2_ | τ_2_(ns) |
| --- | --- | --- | --- | --- |
| w/o crosslinker | 0.765 | 31.94 | 0.211 | 1436 |
| CL-C2 | 0.836 | 23.74 | 0.205 | 917.6 |
| CL-C11 | 1.027 | 14.52 | 0.253 | 746.4 |
| CL-TEG | 0.305 | 121.7 | 0.350 | 891.0 |


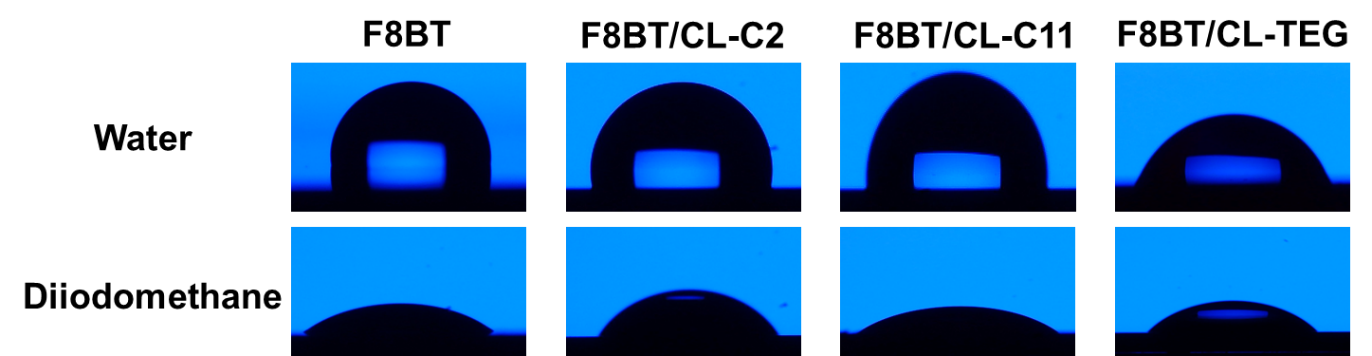


**Figure S22**. Water and diiodomethane contact angle on various surface of F8BT pristine film, and F8BT thin films photocrosslinked with CL-C2, CL-C11, and CL-TEG, respectively.

**Table S5.** Measured contact angles of water and diiodomethane on various surfaces and calculated surface energy values.

|  | *θ*_water_ (**°**) | *θ*_diiodomethane_ (**°**) | *γ*_total_ (mJ m^−2^) |
| --- | --- | --- | --- |
| F8BT | 96.5 | 34.2 | 44.6 |
| F8BT:CL-C2 | 94.6 | 42.6 | 41.5 |
| F8BT:CL-C11 | 93.5 | 38.1 | 43.9 |
| F8BT:CL-TEG | 79.3 | 40.1 | 51.1 |

**Molecular Dynamics (MD) Simulations**

*11. Force Field Parameters and Preparation of Equilibrated Structure of Photocrosslinked Polymers*

The F8BT polymer photocatalyst and photo-crosslinkers (CL) were modeled with OPLS-AA^[S7]^ and additional force field parameters based on the OPLS-AA scheme^[S8-S11]^, employing all-atomistic representations. The SPC/E^[S12]^ model was used for water. For the molecular models of CLs, the diazirine groups were replaced as hydrogens, letting the moiety as 2,2,2-trifluoroethyl group as illustrated in **Figure S18**. This omission was applied because the diazirine groups are replaced by C-C bonds with nearby alkyl groups during the photo-crosslinking reaction, and our simulations targeted the polymer matrix systems after the crosslinking. In **Table S3**, the references for atomic point charges, intramolecular and Lennard-Jones (LJ) interaction parameters for each chemical unit in F8BT and CL are listed. The LJ and intramolecular parameters were taken from the previous simulation studies of corresponding chemical species. The atomic point charges were directly taken from the references when the molecular structures were locally consistent to our model. For the modified 4-(2,2,2-trifluoroethyl)benzonate unit in CL, the atomic point charges were obtained in this work by the restrained electrostatic potential (RESP) fitting method^[S13]^ (**Figure S18** and **Table S4**). The RESP calculations were performed in HF/6-31G* level of theory at the molecular geometries optimized in MP2/6-31G* level of theory, using Gaussian 16 software^[S14]^.

The dihedral rotation of the junction between F8 and BT monomers in the F8BT copolymer chain crucially affects the chain conformations and their packing structures in the matrix. For proper representations of the torsional angles and distributions in simulations, we refined the force field parameters associated with the dihedral rotation at the F8-BT junction. The torsional energy profile calculated by plane-wave density functional theory (PW DFT) with PBE functional in the work by Gmucová et al.^[S15]^ was referred as the target. We fit the coefficients for the Ryckaert-Belleman function (Eq. 1) to match the rotational potential energy along molecular-mechanics (MM) relaxed scan with the DFT energy profile (**Figure S19** and **Table S5**). We leave the torsional potential energy formula below:

$V_{torsion}(\varphi_{ijkl})=\sum_{n=0}^{5} C_{n}{(cos(\psi))}^{n}$ (1)

where *C_n_* notate the Ryckaert-Belleman coefficients, and the transposed dihedral angle ψ = φ − 180°.

**
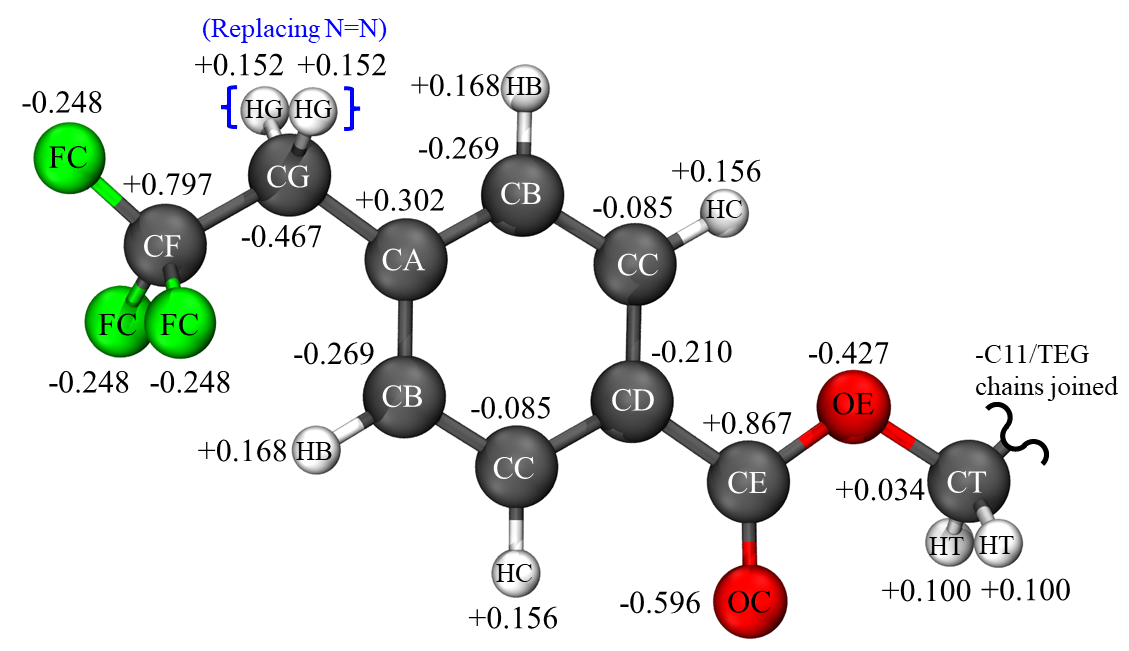
**

**Figure S23**. The modified 4-(2,2,2-trifluoroethyl)benzonate representation for simulations of the photo-crosslinker (CL) moiety, shown except the TEG or C11 bridges. The blue parentheses notate the applied modifications replacing the azirine by hydrogen atoms. The atomic point charges obtained by RESP method are shown with corresponding atom types.

**Table S6**. Summary of references for the OPLS-AA based force field parameters for the chemical units in F8BT polymer chain and crosslinkers (CL) used in simulations in this study. ^a^The torsional parameters at the junction between F8 and BT monomers were reparametrized in this work.

| Unit | Point Charges  Ref. | LJ  Ref. | Intramolecular  Ref. |
| --- | --- | --- | --- |
| F8 | [S6] | [S6] | [S6]^a^ |
| BT | [S7] | [S7] | [S6]^a^ |
| Benzene (CL) | This work | [S5] | [S5] |
| Ester (CL) | This work | [S8] | [S8] |
| CF_3_CH_2_- (CL) | This work | [S9] | [S9] |
| TEG (CL-TEG) | [S5] | [S5] | [S5] |
| C11 (CL-C11) | [S5] | [S5] | [S5] |

**Table S7**. The RESP atomic point charges of the CL molecule except the linked TEG or C11 bridges obtained in this work.

| Atom Type | q (e) | Atom Type | q (e) | Atom Type | q (e) |
| --- | --- | --- | --- | --- | --- |
| CF | +0.797 | CB | −0.269 | CE | +0.867 |
| FC | −0.248 | HB | +0.168 | OC | −0.596 |
| CG | −0.467 | CC | −0.085 | OE | −0.427 |
| HG | +0.152 | HC | +0.156 | CT | +0.034 |
| CA | +0.302 | CD | −0.210 | HT | +0.100 |


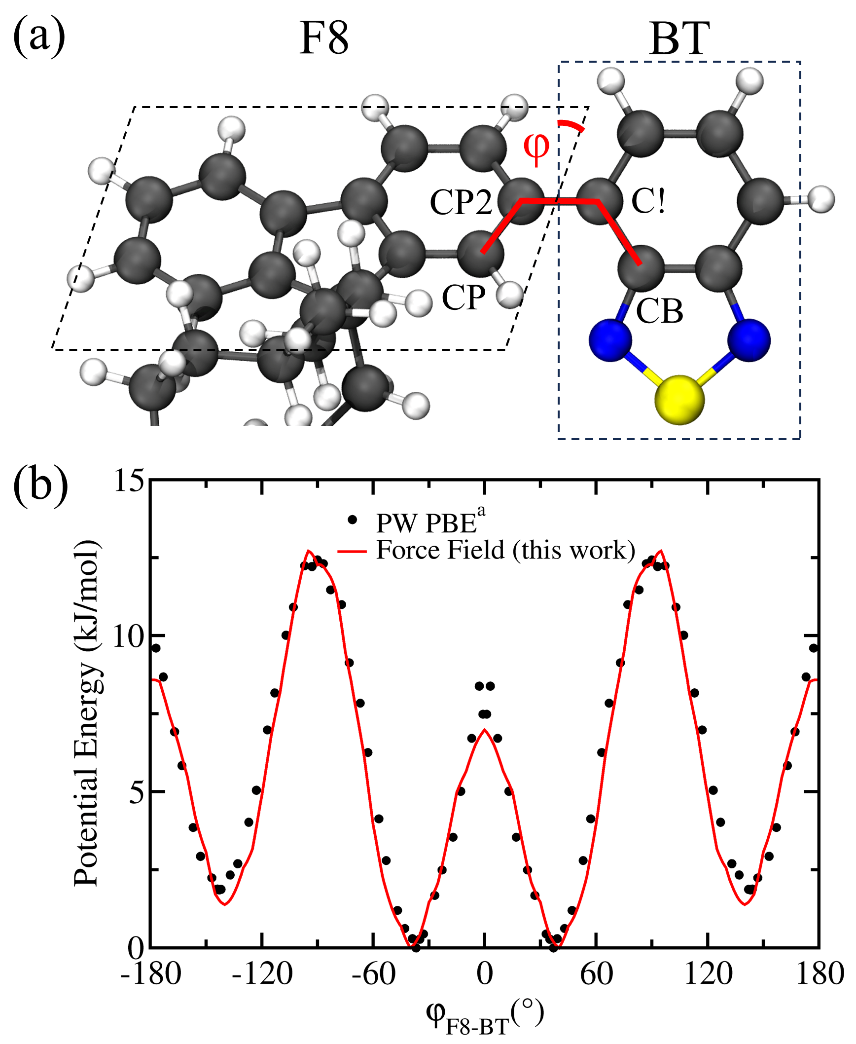


**Figure S24**. (a) The dihedral at the F8-BT junction where torsional force field parameters were refined in this work. The letter symbols on the carbon atoms denote the atom types. (b) Torsional potential energy profiles of the F8-BT junction. ^a^The black dots show the calculated energies by plane-wave density functional theory (PW DFT) with PBE functional in the work by Gmucová et al.^[S14]^

**Table S8**. The Ryckaert-Bellman torsional force field parameters for the F8-BT junction refined in this work (kJ/mol).

| Dihedral Type | C_0_ | C_1_ | C_2_ | C_3_ | C_4_ | C_5_ |
| --- | --- | --- | --- | --- | --- | --- |
| CP-CP2-C!-CB  (F8-BT junction) | 17.9282 | −0.3441 | −46.7519 | 0.0466 | 32.1082 | −0.0396 |

Molecular dynamics (MD) simulations were conducted using OpenMM 8.0 software^[S14]^ with NVIDIA RTX 3090 GPUs. We employed a Langevin thermostat^[S15]^ with a friction coefficient of 1 ps^−1^ and a Monte Carlo barostat^[S18-S19]^ to control the temperature and pressure, respectively. The cutoff distance for Lennard-Jones and short-range electrostatic interactions was set to 1.4 nm. The long-range electrostatic interactions were computed by the particle mesh Ewald (PME)^[S20]^ method. The timestep of simulations was 2 fs, while the C-H bonds of all molecules and water molecular geometries were kept rigid.

We constructed the initial configurations of the polymer matrix systems by PACKMOL^[S21]^ software, randomly locating 50 (F8BT)_10_ copolymer chains, 100 CL-TEG or CL-C11 crosslinker molecules, and 9452 water molecules in arbitrarily large cubic boxes. The CL molecules were not covalently linked with the (F8BT)_10_ chains at this initial stage of MD simulations. The degree of polymerization *N* = 10 for the (F8BT)_10_ copolymer chain was selected to let the chain length scale affordable to the capability of atomistic MD simulations in nanoscale, while avoiding misrepresentations of the chain packing behaviors by embodying too short oligomers. The molar ratio of 5:1 for F8BT co-monomers to CL molecules corresponds to the experimental mass ratio of 10:3, since the molar masses of F8BT co-monomer and CL-TEG are 529 g/mol and 762 g/mol, respectively. The number of water molecules corresponds to 33.3 wt% of the entire polymer matrix systems.

The intense steric hindrance of the polymer chains makes the dynamics of the polymer matrix systems highly trapped. To properly represent the microscopic wetting behaviors of F8BT and CL moieties by water in MD simulations, the chain packing behaviors in the polymer matrix should be sampled through tailored equilibration protocols. The equilibration protocol should allow the molecules to travel across the system box, adjust their conformations enough to remove the transient vacant spaces among highly entangled chains with reaching the most efficient packing. Therefore, we applied a specific stepwise equilibration protocol as described below.

The systems underwent an initial density equilibration in an NPT ensemble with T = 300 K and P = 1 atm for 25 ns. To represent the difference of the amount of water penetrated into the polymer matrices depending on water affinity of the crosslinkers, we removed excess water molecules except the ones within 3.5 Å of the TEG or C11 bridges. After removing these excess water molecules, 1199 and 603 water molecules were kept remaining in the polymer matrices with CL-TEG and CL-C11 crosslinkers, respectively. Since the water removal creates cavities in the systems, an additional stepwise re-equilibration protocol was proceeded as follows:

1. Density equilibration in NPT ensemble at T = 300 K and P = 1 atm for 20 ns
2. Annealing in NVT ensemble at T = 1000 K for 20 ns
3. High-pressure density equilibration in NPT ensemble at T = 300 K and P = 10 atm for 20 ns
4. Annealing in NVT ensemble at T = 1000 K for 10 ns
5. High-pressure density equilibration in NPT ensemble at T = 300 K and P = 10 atm for 20 ns

Once the polymer matrix systems with blended (F8BT)_10_ copolymer chains, unlinked CL molecules and water were equilibrated, we applied an intermediate covalent crosslinking between F8 alkyl groups and CL molecules prior to the next stages of MD simulations (**Figure S20**). This step is in line with the experiment protocol in this study where the photo-crosslinking reactions occurred after emulsifications of the solutions of polymer photocatalysts with CL molecules. In the last snapshot of the equilibrated polymer matrix, as illustrated in **Figure S25**, for each CG carbon atom of the CL molecules, the closest carbon atom in the F8 alkyl groups was selected as the cross-linking target. Selecting the same F8 alkyl carbon atom by multiple CL carbon atoms was avoided. For these selected C-C crosslink atomic pairs, we applied an additional harmonic potential of force constant *k* = 22426 kJ/mol/nm^2^ and equilibrium bond length *r_0_* = 1.529 Å to embody the newly formed C-C covalent bonds. As a result of this computational crosslinking, all kinds of carbon positions from the root to end of F8 alkyl chain participated meaningfully in crosslinking, as shown in the probability distribution in **Figure S26**.

The photocrosslinked polymer matrices were further equilibrated in NPT ensemble at T = 300 K and P = 1 atm for 20 ns, and in NVT ensemble at T = 300 K for 20 ns. Production runs of 500 ns in NVT ensemble at T = 300 K were followed.


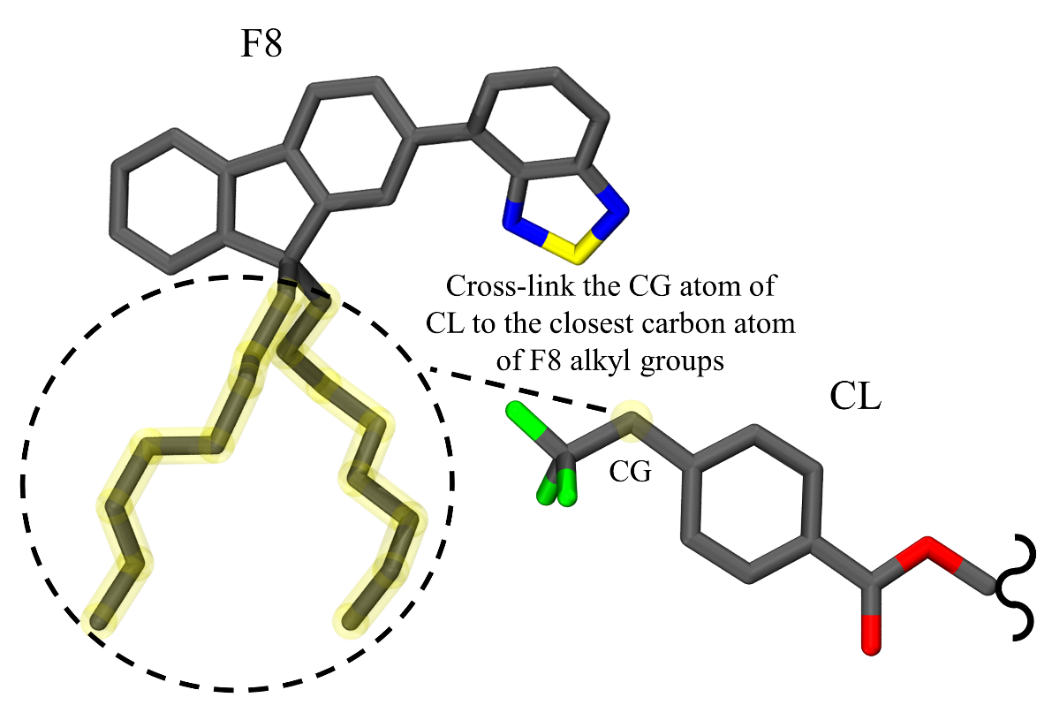


**Figure S25**. Schematics of the hypothetical C-C crosslink search in MD simulations. Hydrogen atoms are omitted for clarity. The yellow-marked carbon atoms in F8 alkyl side chains and the 2,2,2-trifluoroethyl group in CL molecules were the potential linkage targets. For each CG carbon atom in the CL molecules, the closest carbon atom which belongs to the alkyl side chains of any F8 monomer was selected. MD simulations were continued with applying covalent bonds additionally on these C-C pairs.


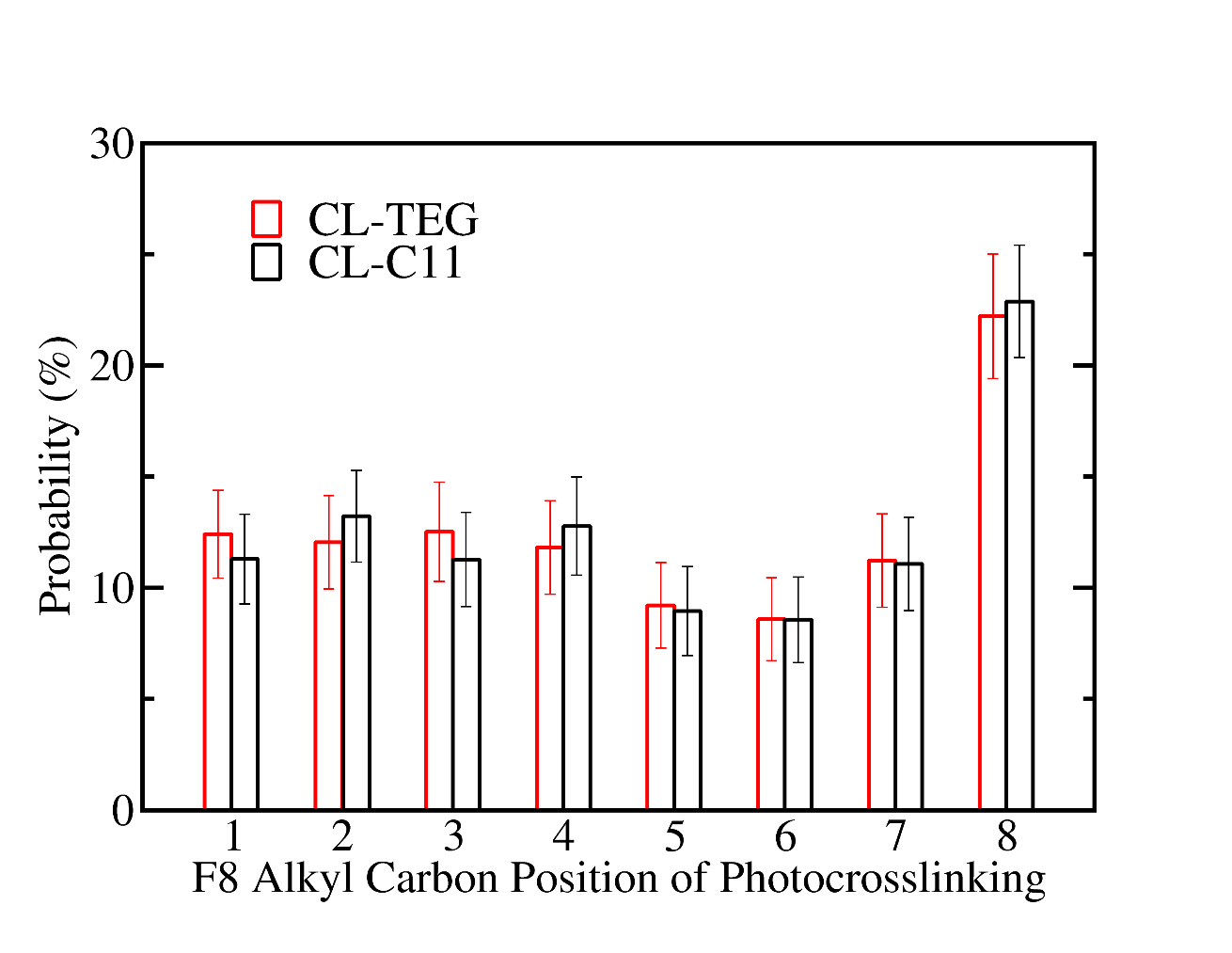


**Figure S26**. Histogram of the carbon position of photocrosslinking to the F8 alkyl side chains in MD simulations. These were predicted by searching the closest F8 alkyl carbon atoms from the expected photo-generated carbene site of the crosslinkers, as depicted in **Figure S22**. The carbon position indices of 1 and 8 indicate the root and end of the alkyl side chain, respectively.

*12. MD Simulations of Photocrosslinked Polymers with Computational Exciton Generation*

After the crosslinked polymer matrix systems were equilibrated, we evaluated the ability of water access to the F8BT copolymer chains when the photo-induced excitons are formed. We approximated the excitons as localized net charges of +1 e on a F8 monomer unit and −1 e on a BT monomer unit, respectively. This approximation was set considering the previous study by Dey et al.^[S20]^ which reported localization of the LUMO (lowest unoccupied molecular orbital) of F8BT co-monomer on the BT unit by DFT calculations. As shown in **Figure S27**, for the MD simulations of polymer matrices with photo-induced excitons on F8BT polymer chains, we applied adjustments on the point charges of atoms participating in conjugations for randomly selected 20 percent of adjacent F8-BT co-monomers in the systems. The charge adjustment values were determined by taking differences between the RESP atomic charges of the +1 charged cation of the F8 unit or the −1 charged anion of the BT unit and their neutral forms (**Table S8**).

After applying the computational exciton generation by atomic charge modification as explained above, the polymer matrix systems were equilibrated again in NPT ensemble at T = 300 K and P = 1 atm for 20 ns, and then in NVT ensemble at T = 300 K for 20 ns. Next, production runs of 500 ns in NVT ensemble at T = 300 K were performed.


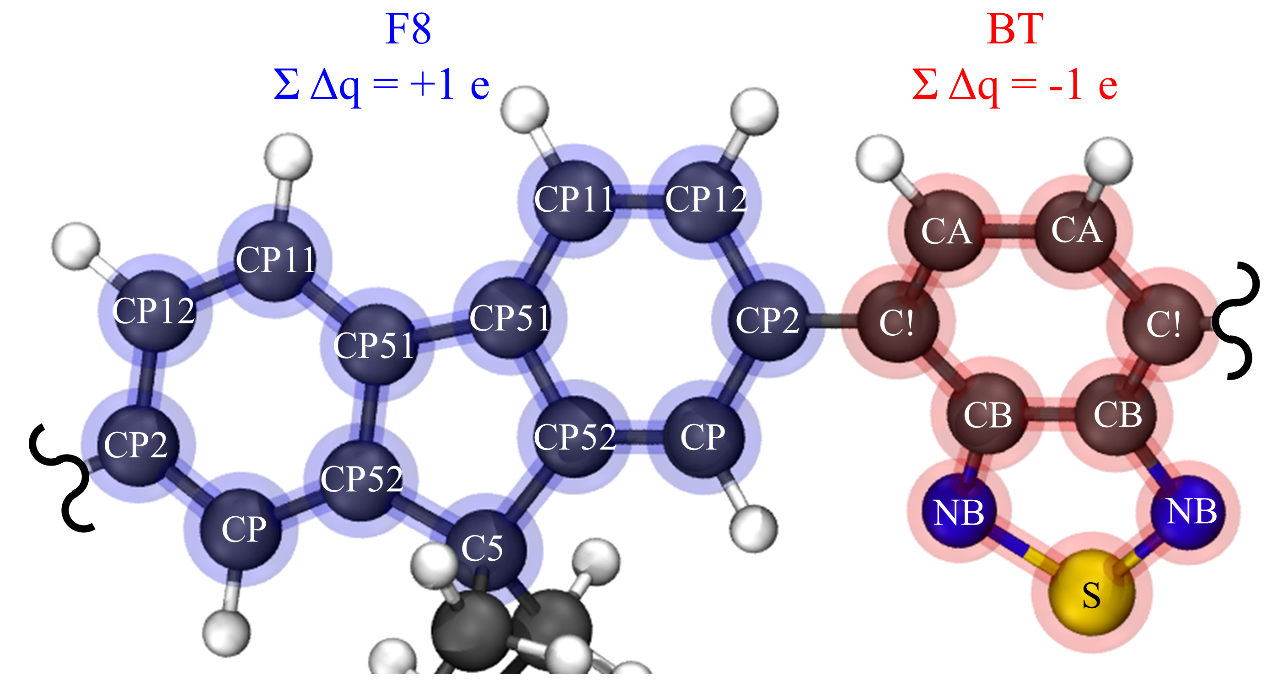


**Figure S27**. Schematics of the hypothetical exciton generation on F8BT co-monomers in MD simulations. For randomly selected 20 percent of adjacent F8 and BT co-monomers in the system, the atomic partial charges were adjusted. For the blue-marked atoms in F8, atomic point charge adjustments with total of +1 e per monomer were applied. For the red-marked atoms in BT, atomic point charge adjustments with total of −1 e per monomer were applied. The detailed adjustment values are listed in Table S4.

**Table S9**. The atomic point charge adjustments on the hypothetical F8BT co-monomer excitons in MD simulations.

| F8 (Σ Δq = +1 e) | | BT (Σ Δq = −1 e) | |
| --- | --- | --- | --- |
| Atom Type | Δq (e) | Atom Type | Δq (e) |
| CP | +0.001 | S | +0.248 |
| CP11 | +0.065 | NB | −0.736 |
| CP12 | +0.044 | CB | +0.029 |
| CP2 | +0.211 | C! | +0.291 |
| CP51 | +0.102 | CA | −0.208 |
| CP52 | −0.054 |  |  |
| C5 | +0.262 |  |  |

*13. Radial and Spatial Distributions from MD Simulation Trajectories*

The water coordination on the photocrosslinker bridges and F8BT backbones was quantified by radial distribution functions (RDFs) and spatial distribution functions (SDFs) of water oxygen atoms around specified reference atoms in the crosslinkers or F8BT units. The distribution functions were computed from the MD simulation trajectories of production runs.

The RDFs were calculated using MDTraj^[S23]^ python libraries. In **Figure S28**, the RDFs of water O atoms with respect to CL-TEG O atoms and CL-C11 C atoms in photocrosslinked polymer matrices before computational exciton generation are drawn. **Figure S29** illustrates the corresponding snapshot of hydration shells around the crosslinker bridge. The comparisons of RDFs before and after the computational exciton generation in the CL-TEG linked polymer matrix are presented in **Figure S30**.

The SDFs were calculated by TRAVIS^[S24]^ software using the following local coordinate definitions: for CL-TEG, O atom as origin and C-O-C plane as the reference xy-plane; for CL-C11, in each (CH_2_)_3_ moiety, the middle C atom as origin and C-C-C plane as the reference xy-plane; for F8 unit, the C5 carbon atom as origin and the conjugated molecular plane as the reference xy-plane; for BT unit, the S atom as origin and the conjugated molecular plane as the reference xy-plane. For data presentations, we computed two-dimensionally projected SDFs by taking the average local number density of water O atoms within 3.0 Å below and above the reference molecular xy-plane of the SDFs.


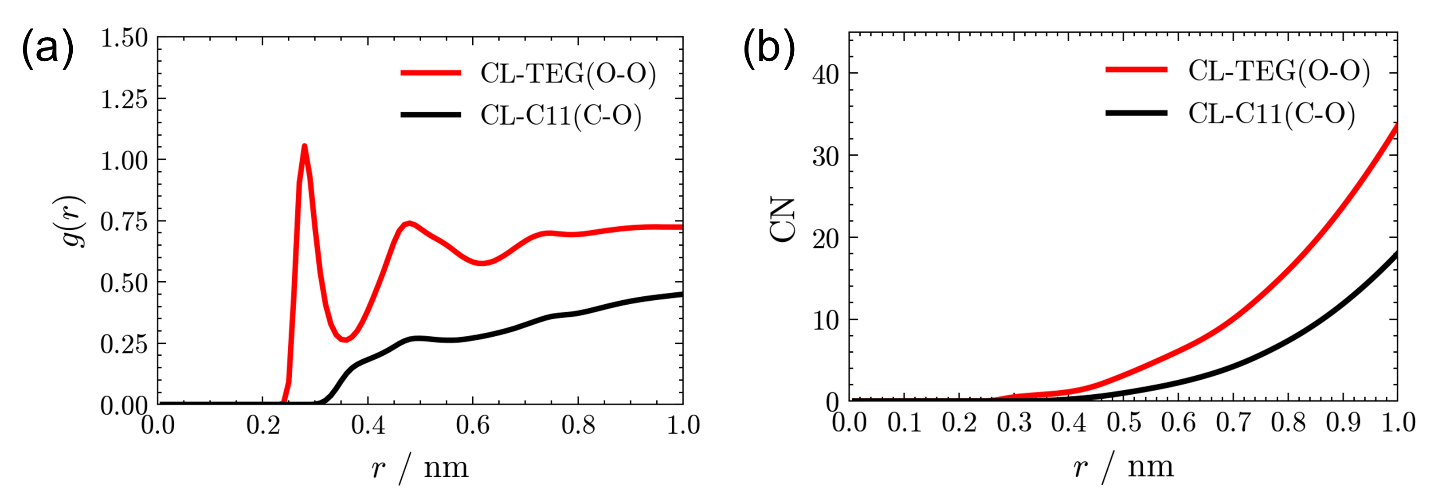


**Figure S28**. (a) Radial distribution functions (RDFs) and (b) cumulative coordination numbers of water O atoms on CL-TEG O atoms and CL-C11 C atoms predicted in MD simulations.


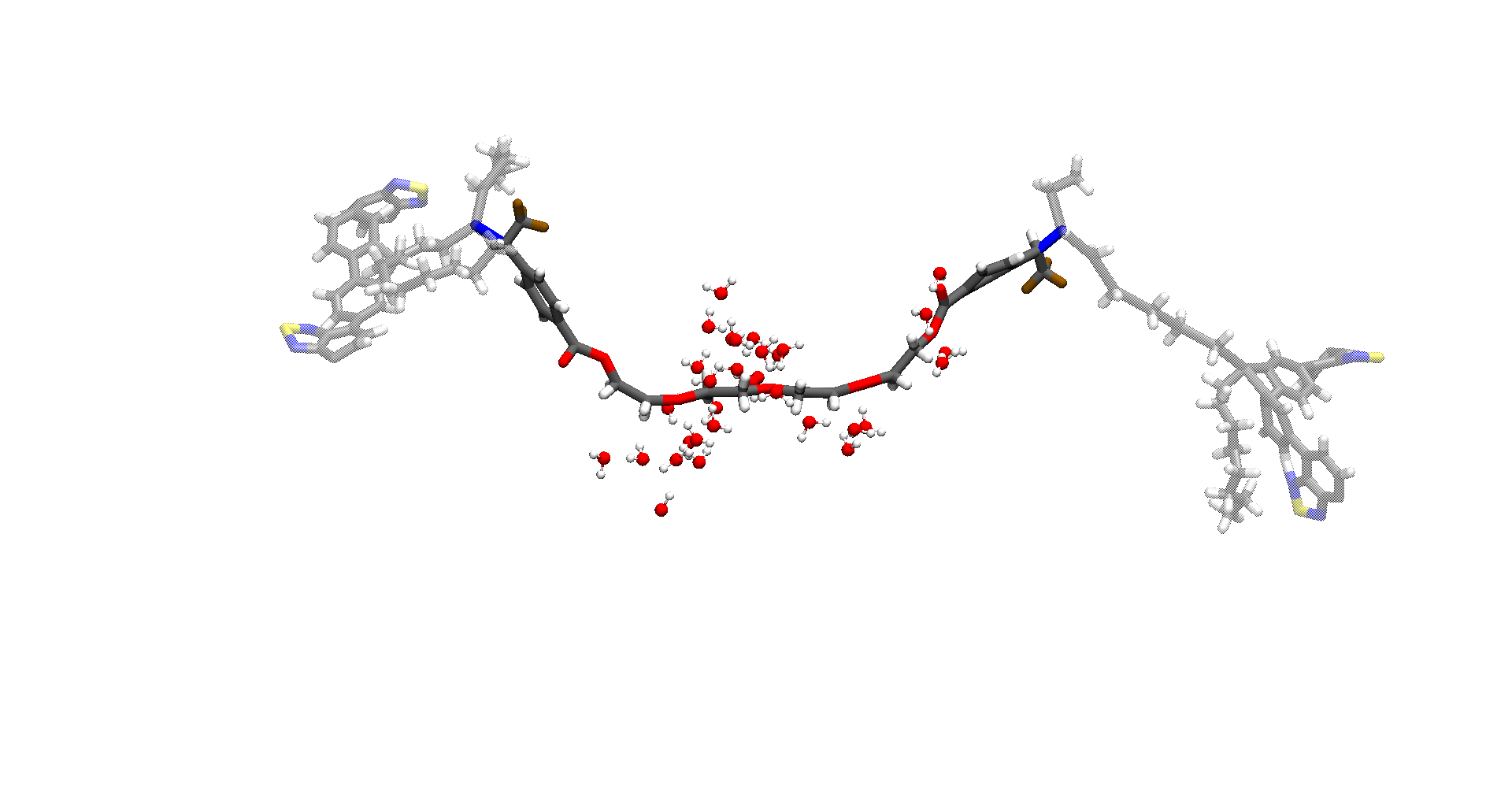


**Figure S29**. Snapshot of representative hydration shells on the TEG bridge oxygen in MD simulations.


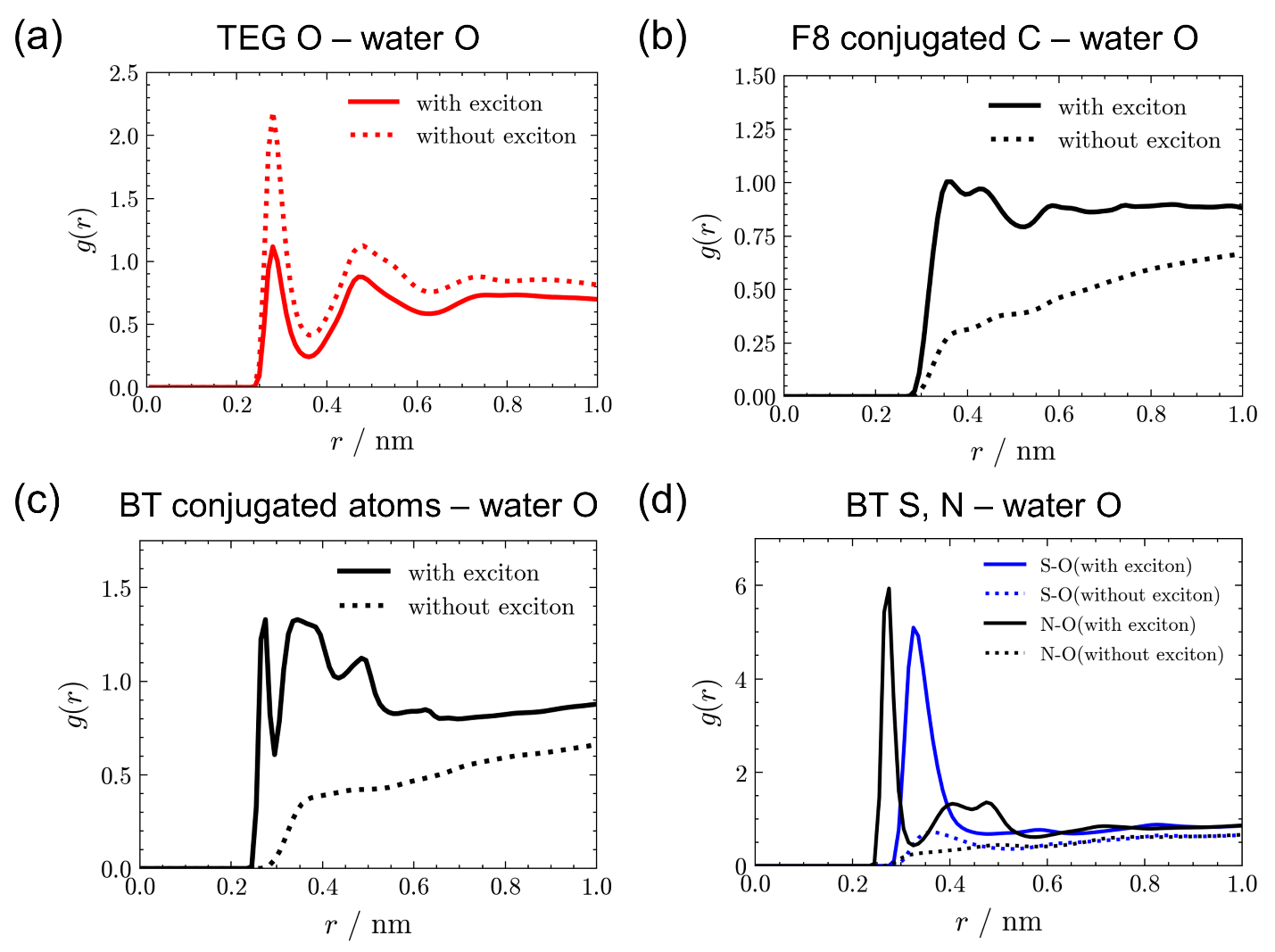


**Figure S30**. Comparisons of RDFs of water O atoms before and after exciton generation on randomly chosen 20 percent of F8BT copolymer units in the MD simulation of the F8BT photocrosslinked by CL-TEG. The RDFs are drawn for the following atomic pair groups: (a) TEG O – water O, (b) F8 conjugated C – water O, (c) BT conjugated N, S, C – water O, and (d) combined plot of BT S – water O, BT N – water O.


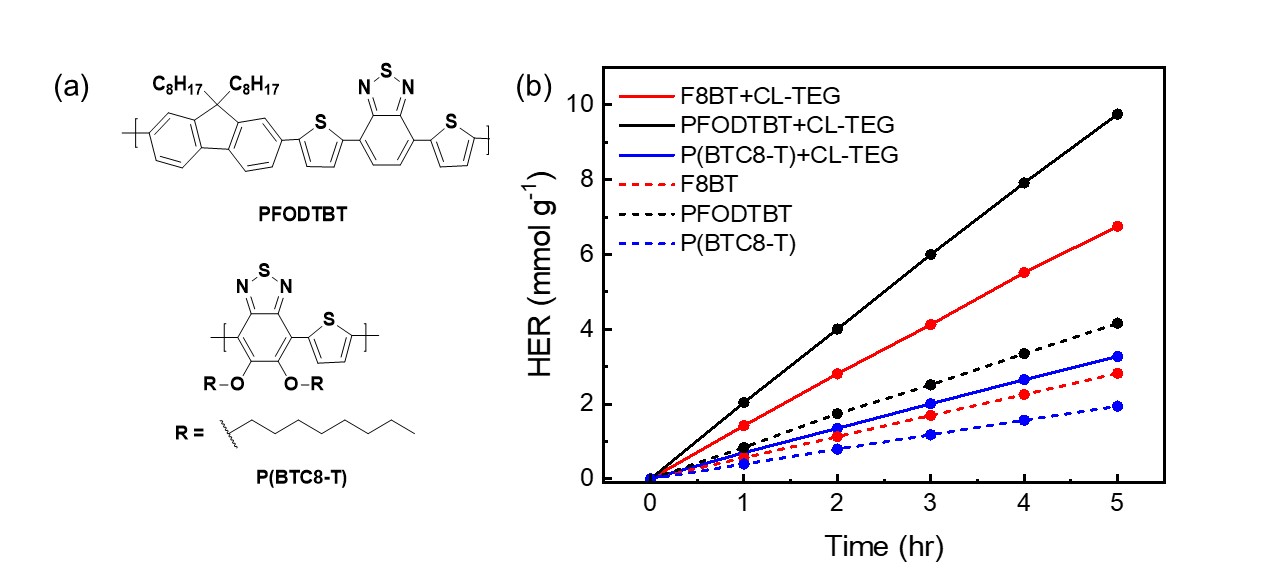


**Figure S31**. (a) The chemical structures of PFODTBT and P(BTC8-T). (b) Comparison of hydrogen evolution performance between the photocrosslinked conjugated polymers with CL-TEG and without photocrosslinker agents, respectively.

**References**

[S1] Sakurai T. Yasui, S. Mizuno, *Asian J. Org. Chem.* **2015**, 4, 724–728.

[S2] S. An, Z. Wu, H. Jeong, J. Lee, S. Y. Jeong, W. Lee, S. Kim, J. W. Han, J. Lim, H. Cha, H. Y. Woo, D. S. Chung, *Small* **2023**, 19, 2204905.

[S3] J. M. Yuk, J. Park, P. Ercius, K. Kim, D. J. Hellebusch, M. F. Crommie, J. Y. Lee, A. Zettl, A. P. Alivisatos, *Science* **2012**, *336*, 61

[S4] M. Qureshi, K. Takanabe, *Chem. Mater.* **2017**, 29, 158

[S5] Jeremy E. Wulff et al., Structure–function relationships in aryl diazirines reveal optimal design features to maximize C–H insertion, *Chem. Sci.* **2021**, 12, 12138.

[S6] W. Regan, N. Alem, B. Alemán, B. Geng, Ç. Girit, L. Maserati, F. Wang, M. Crommie, A. Zettl, *Appl. Phys. Lett.* **2010**, *96*, 113102

[S7] Jorgensen, W. L.; Maxwell, D. S.; Tirado-Rives, J. Development and Testing of the OPLS All-Atom Force Field on Conformational Energetics and Properties of Organic Liquids. *J. Am. Chem. Soc.* **1996**, *118*, 11225-11236.

[S8] Wildman, J.; Repiscak, P.; Paterson, M. J.; Galbraith, I. General Force-Field Parametrization Scheme for Molecular Dynamics Simulations of Conjugated Materials in Solution. *J. Chem. Theory Comput.* **2016**, *12*, 3813-3824.

[S9] Gertsen, A. S.; Sørensen, M. K.; Andreasen, J. W. Nanostructure of organic semiconductor thin films: Molecular dynamics modeling with solvent evaporation. *Phys. Rev. Mater.* **2020**, *4*, 075405.

[S10] Price, M. L.; Ostrovsky, D.; Jorgensen, W. L. Gas‐Phase and Liquid‐State Properties of Esters, Nitriles, and Nitro Compounds with the OPLS‐AA Force Field. *J. Comput. Chem.* **2001**, *22*, 1340-1352.

[S11] Lachet, V.; Teuler, J.-M.; Rousseau, B. Classical Force Field for Hydrofluorocarbon Molecular Simulations Application to the Study of Gas Solubility in Poly(vinylidene fluoride). *J. Phys. Chem. A* **2015**, *119*, 140−151.

[S12] Berendsen, H. J.; Grigera, J. R.; Straatsma, T. P. The Missing Term in Effective Pair Potentials. *J. Phys. Chem.* **1987**, *91*, 6269-6271.

[S13] Bayly, C. I.; Cieplak, P.; Cornell, W.; Kollman, P. A. A Well-Behaved Electrostatic Potential Based Method using Charge Restraints for Deriving Atomic Charges: the RESP Model. *J. Phys. Chem.* **1993**, *97*, 10269-10280.

[S14] Frisch, M. J.; Trucks, G. W.; Schlegel, H. B.; Scuseria, G. E.; Robb, M. A.; Cheeseman, J. R.; Scalmani, G.; Barone, V.; Petersson, G. A.; et al., *Gaussian 16, Rev.C.01*, Gaussian, Inc.: 2016.

[S15] Gmucova, K.; Konopka, M.; Vegso, K.; Bokes, P.; Nadazdy, V.; Vary, T. Correlation between Molecular Stereostructure, Film Microstructure, and Electronic Structure of Polyfluorene and Fluorene Based Alternating Copolymers F8BT and PFO–DBT. *J. Phys. Chem. C*, **2021**, *125*, 8045-8054.

[S16] Eastman, P.; Swails, J.; Chodera, J. D.; McGibbon, R. T.; Zhao, Y.; Beauchamp, K. A.;

Wang, L.-P.; Simmonett, A. C.; Harrigan, M. P.; Stern, C. D.; et al. OpenMM 7: Rapid Development of High Performance Algorithms for Molecular Dynamics. *PLoS Comput. Biol.* **2017**, *13*, e1005659.

[S17] Izaguirre, J. A.; Sweet, C. R.; Pande, V. S. Multiscale Dynamics of Macromolecules Using Normal Mode Langevin. *Pac. Symp. Biocomput.* **2009**, *15*, 240–251.

[S18] Chow, K.-H.; Ferguson, D. M. Isothermal-Isobaric Molecular Dynamics Simulations with Monte Carlo Volume Sampling. *Comput. Phys. Commun.* **1995**, *91*, 283–289.

[S19] Aqvist, J.; Wennerstrom, P.; Nervall, M.; Bjelic, S.; Brandsdal, B. O. Molecular Dynamics Simulations of Water and Biomolecules with a Monte Carlo Constant Pressure Algorithm. *Chem. Phys. Lett.* **2004**, *384*, 288–294.

[S20] Essmann, U.; Perera, L.; Berkowitz, M. L.; Darden, T.; Lee, H.; Pedersen, L. G. A Smooth Particle Mesh Ewald Method. *J. Chem. Phys.* **1995**, *103*, 8577–8593.

[S21] Martinez, L.; Andrade, R.; Birgin, E. G.; Martinez, J. M. PACKMOL: A Package for Building Initial Configurations for Molecular Dynamics Simulations. *J. Comput. Chem.* **2009**, *30*, 2157–2164.

[S22] Dey, A.; Chandrasekaran, N.; Chakraborty, D.; Johari, P.; McNeill, C. R.; Rao, A.; Kabra, D. Kinetics of thermally activated triplet fusion as a function of polymer chain packing in boosting the efficiency of organic light emitting diodes. *npj Flexible Electron.* **2018**, *2*, 28.

[S23] McGibbon, R.T.; Beauchamp, K.A.; Harrigan, M. P.; Klein, C.; Swails, J. M.; Hernández, C. X.; Schwantes, C. R.; Wang, L. P.; Lane, T. J.; Pande, V. S. MDTraj: a Modern Open Library for the Analysis of Molecular Dynamics Trajectories. *Biophys. J.* **2015**, *109*, 1528-1532.

[S24] Brehm, M.; Thomas, M.; Gehrke, S.; Kirchner, B. TRAVIS—A Free Analyzer for Trajectories from Molecular Simulation. *J. Chem. Phys.* **2020**, *152*, 164105.
